# PINK1/Parkin promotes liver regeneration via Sigma-1 ubiquitination to inhibit ER-Mitochondrial calcium transfer

**DOI:** 10.7150/thno.115726

**Published:** 2026-01-01

**Authors:** Jian Xu, Yuechen Wang, Weizhe Zhong, Haoran Hu, Ye Zhang, Yiyun Gao, Ping Wang, Zhuqing Rao, Haoming Zhou, Xuehao Wang

**Affiliations:** 1 Hepatobiliary/Liver Transplantation Center, The First Affiliated Hospital of Nanjing Medical University, China.; 2 Key Laboratory of Liver Transplantation, Chinese Academy of Medical Sciences, China.; 3 NHC Key Laboratory of Living Donor Liver Transplantation, Nanjing Medical University, China.; 4 Department of Anesthesiology, The First Affiliated Hospital of Nanjing Medical University, China.

**Keywords:** liver regeneration, PINK1/Parkin, sigma-1, MAM calcium channel, succinate

## Abstract

**Rationale:** Liver regeneration is regulated by both metabolic processes and immune responses. Nonetheless, there is limited comprehension of the mechanisms involved. PINK1/Parkin-mediated mitophagy has been well documented, the role and underlying alternative mechanism of PINK1/Parkin in regulating mitochondrial metabolism during liver regeneration remains unclear.

**Methods:** Liver tissues from mice undergoing hepatectomy were utilized to evaluate the expression levels of PINK1/Parkin. Hepatocyte-specific PINK1 knockout and transgenic mouse models were generated to investigate the impact of PINK1 on regeneration. Mass spectrometry, co-immunoprecipitation, and ubiquitination assays were performed to explore the underlying molecular mechanisms.

**Results:** We observed PINK1/Parkin expression was markedly upregulated in hepatic tissue following liver resection. PINK1 depletion in hepatocytes caused impaired liver regeneration. Moreover, mitochondrial calcium overload was found be responsible for restricted TCA by inhibiting succinate dehydrogenase activity in PINK1 deficient hepatocytes. Interestingly, PINK1 deficiency leads to succinate accumulation and release from hepatocytes, which impairs liver regeneration by restricting macrophage pro-repair phenotypes. This effect was further confirmed by enhanced regeneration in myeloid SUCNR1 knockout mice. Mechanistically, Sigma-1 is a molecular chaperone of the endoplasmic reticulum calcium channel IP3R, which helps maintain its normal functional conformation. Parkin was able to bind Sigma-1 through its UBL domain, facilitating its k48-linked ubiquitination, which promotes Sigma-1 degradation and subsequently suppressing calcium transfer from the ER to mitochondria at the mitochondrial-associated ER membrane.

**Conclusions:** Collectively, PINK1/Parkin signaling regulates hepatocellular mitochondrial ATP and succinate production by modulating ER-mitochondria calcium transfer to promote liver regeneration, revealing a promising therapeutic target for liver regeneration.

## Introduction

The liver is a unique organ with diverse functions, including metabolism, detoxification, immune response, and coagulation. It is particularly notable for its remarkable regenerative capacity, enabling restoration of both mass and function following surgical resection, viral injury, or toxic exposure, thereby re-establishing systemic homeostasis 1. Following liver injury, residual hepatocytes re-enter the cell cycle and rapidly undergo mitosis to generate new hepatocytes. Under normal physiological conditions, hepatocyte proliferation supports ongoing liver function 2. However, during hepatic stress, compensatory replication of surviving hepatocytes, along with contributions from other cell types such as liver progenitor cells and cholangiocytes, participates in the restoration of hepatic architecture and function 3-6. Numerous signaling molecules regulate cell proliferation during liver regeneration, with hepatocyte metabolic processes and growth factors, including hepatocyte growth factor and mesenchymal-epithelial transition factor, playing pivotal roles. Thus, elucidating the molecular and signaling mechanisms underlying liver regeneration may offer novel therapeutic strategies for restoring liver function in clinical settings.

Mitochondria play an essential role in the life cycle of various cell types 7. As the central hub for cellular energy metabolism, mitochondria are involved in key metabolic processes, including ATP production 8, β-oxidation of fatty acids 9, and gluconeogenesis, with the tricarboxylic acid (TCA) cycle being central to both energy production and metabolism. Mitochondria also generate reactive oxygen species (ROS) as byproducts of the electron transport chain, serving as substrates for anabolic processes and signaling 10, 11. Succinate, a key metabolite in the TCA cycle, has emerged as a critical regulator of cellular nutrient metabolism, prompting extensive research into its functional roles. When mitochondrial function is impaired, succinate accumulates, disrupting the TCA cycle 12. This accumulated succinate can be converted into immunoregulatory signals via autocrine or paracrine mechanisms, playing crucial roles in inflammation, tissue injury, and even tumor modulation 13-15. Although the direct pathogenic role of succinate accumulation remains unclear, it may accelerate disease progression 16. Even mild mitochondrial dysfunction can disrupt cellular metabolism and redox balance, impair mitochondrial calcium homeostasis, and lead to cellular dysfunction 17, 18. PINK1/Parkin are critical effectors of mitochondrial quality control. PINK1, a kinase imported into mitochondria, typically translocate to the inner mitochondrial membrane and is degraded by mitochondrial proteases under normal conditions 19, 20. Upon mitochondrial membrane potential loss, PINK1 accumulates on the outer mitochondrial membrane and recruits the E3 ubiquitin ligase Parkin, which then ubiquitinates outer mitochondrial membrane proteins 21. This process triggers the proteasomal degradation of outer mitochondrial membrane proteins and facilitates selective mitochondrial autophagy. Despite this, the role of PINK1 in liver regeneration and its associated mechanisms remains inadequately explored.

Sigma receptors, non-opioid proteins located in the endoplasmic reticulum (ER), exist in two subtypes: Sigma-1 and Sigma-2 22. The sequence of Sigma-2 remains unidentified. Sigma-1 is primarily localized to the membrane structures of the mitochondria-associated ER membrane (MAMs), where it functions as a molecular chaperone to stabilize the inositol 1,4,5-trisphosphate receptor type 3 (IP3R), thereby facilitating calcium transfer from the ER to mitochondria 23. Studies suggest that Sigma-1 in MAMs is also a substrate for ER-associated protein degradation (ERAD), mediating its ubiquitination through the Sel1L-Hrd1 complex to regulate mitochondrial dynamics 24. While Sigma-1's involvement in mitochondrial regulation via MAMs suggests its role in mitochondrial function, the exact molecular mechanisms by which it participates in liver regeneration remain unclear.

In this study, we identify PINK1/Parkin as essential regulators in liver regeneration. We show that PINK1/Parkin expression in hepatocytes is significantly upregulated following partial hepatectomy (PHx). Hepatocyte-specific deletion of PINK1 severely impairs liver regeneration post PHx. Mechanistically, as an E3 ubiquitin ligase, Parkin ubiquitinates Sigma-1 during liver regeneration, thereby inhibiting ER-to-mitochondria calcium transfer. In PINK1-deficient hepatocytes, enhanced calcium transfer between the ER and mitochondria results in mitochondrial calcium overload, inhibiting succinate dehydrogenase activity, disrupting the TCA cycle, and leading to succinate accumulation. This accumulation exerts paracrine effects on macrophages, hindering liver regeneration.

## Methods

### Animals

Hepatocyte-specific PINK1 knockout (hKO) mice and wild type control mice (WT) were generated by GemPharmatech Co., Ltd. The PINK1^loxp/loxp^ mice were bred with Albumin-Cre mice to generate hKO mice. Hepatocyte-specific PINK1 overexpression (hKI) mice were generated by CRISPR/Cas9 gene knock-in technology in GemPharmatech Co., Ltd. Myeloid-specific SUCNR1 knockout (hKO) mice were generated by breeding SUCNR1-LoxP with Lyz2-Cre mice (Nanjing Biomedical Research Institute of Nanjing University). The mice were housed in a standard specific pathogen-free environment with a 12 h light/dark cycle. All animals received humane care, and all procedures involving animals were conducted in accordance with relevant legal and ethical requirements, following the protocols (IACUC-2311005) approved by the Institutional Animal Care and Use Committee of Nanjing Medical University.

### 70% partial hepatectomy

Anesthesia was induced with 2-3% isoflurane. A midline abdominal incision was performed, and approximately 70% of the liver (left and central lobes) was resected. After achieving hemostasis, the abdominal cavity was closed. Postoperatively, analgesia was administered with meloxicam (1 mg/kg), and the animals were closely monitored. Tissue specimens were collected at various time points following surgery for subsequent analyses.

### Quantitative real-time-PCR

Total RNA was extracted from cells using TRIzol reagent (Invitrogen) according to the manufacturer's instructions. RNA concentration and quality were evaluated using Nano-drop system and was then reverse-transcribed into cDNA by commercially available kit (Vazyme, Nanjing, China). Quantitative real-time PCR was carried out using SYBR Green (Roche). The expression levels of target genes were normalized to β-actin expression.

### Western blot analysis

Protein of the liver tissue or cells were extracted prepared. Protein samples were resolved by sodium dodecyl sulfate-polyacrylamide gel electrophoresis and transferred to a PVDF membrane for subsequent analysis. Membranes were blocked with blocking buffer for 30 min at RT and then incubated with primary antibody overnight at 4 °C. After 2 h of incubation with the HRP-conjugated secondary antibody, chemiluminescent detection was performed using immobilon ECL Ultra western HRP substrate (Vazyme Biotechnology). Images were captured with a Vilber chemiluminescent imaging system (Vilber). Densitometric analysis was conducted using ImageJ software to assess alterations in protein expression.

### Histopathology and immunohistochemical staining

Liver tissue sections were stained using haematoxylin and eosin (H&E) to observe cell division using light microscopy. Paraffin sections were deparaffinized and rehydrated, followed by antigen retrieval, and then the tissue sections were incubated with the corresponding primary antibodies at 4 °C overnight. The chromogenic reaction was performed using a DAB solution for IHC assays. Primary antibodies were BrdU (Cell Signaling Technology), Ki67 (Cell Signaling Technology), PCNA (Cell Signaling Technology).

### Isolation of liver cells

As previously reported, the liver was perfused in situ through the portal vein with calcium- and magnesium-free Hanks' balanced salt solution supplemented with 2% heat-inactivated FBS, followed by collagenase IV (Sigma-Aldrich). The perfused liver was dissected and filtered through a 70 µM nylon mesh cell strainer (BD Biosciences). Hepatocytes were suspended in 20 mL DMEM containing 10% FBS and separated into parenchymal and non-parenchymal cells as described below. Primary hepatocytes were pelleted by centrifugation at 50 g for 2 min. The cell pellet was resuspended in 20 mL of 50% cold Percoll solution (Sigma) and centrifuged at 150 g for 5 min. The hepatocyte pellet was resuspended in DMEM with 10% FBS and seeded onto type I collagen-coated plates. NPCs were suspended and allowed to adhere to cell culture plates in DMEM supplemented with 10% FBS, incubated at 37 °C for 15 min. Non-adherent cells were removed by replacing the culture medium, and the adherent cells were identified as macrophages.

### Immunofluorescence staining

Primary hepatocytes were fixed with 4% paraformaldehyde, permeabilized with 0.1% Triton X-100, and blocked with 5% BSA. Cells were incubated with primary antibodies overnight at 4 °C. Primary antibodies were PINK1, Parkin, Sigma-1, IP3R1, TOM20, PDI (Cell Signaling Technology). Then, incubated with alexa fluor-conjugated secondary antibodies (Thermo Fisher) for 1 h. Nuclei were stained with DAPI, and images were acquired by confocal microscope. Fluorescence intensity and localization were analyzed using ImageJ.

### Mitochondrial membrane potential assessment

The mitochondrial membrane potential was monitored using the JC-10 assay kit (Yeasen Biotechnology). Briefly, JC-10 is a lipophilic dye that accumulates in healthy mitochondria, forming red-fluorescent J-aggregates. In damaged mitochondria, it forms green-fluorescent J-monomers. A reduction in the red signal observed under a fluorescence microscope indicates a decrease in the mitochondrial membrane potential.

### Oxidative stress detection

MDA, GSH, GSSG, SOD, and ROS levels were measured using commercial kits (Beyotime Biotechnology). Liver tissues were washed with PBS, homogenized in lysis buffer, and sonicated. After centrifugation (10,000×g, 10 min), the supernatant was collected. In addition, MDA, SOD, GSH, GSSG, and ROS levels were measured using a microplate reader and normalized to protein concentration.

### Mitochondrial OCR and ECAR measurement

Agilent Seahorse XF96 analyzer was used to simultaneously measure the rate of oxygen consumption and extracellular acidification from primary hepatocytes. Primary hepatocytes were plated in Seahorse XF96 cell culture plates and allowed to adhere. After adherence, the cells were incubated with the following reagents: 10 mM glucose to stimulate glycolysis, 1 µM oligomycin to inhibit ATP synthase (Sigma-Aldrich), 1 µM FCCP to uncouple mitochondrial respiration (Sigma-Aldrich), 0.5 µM rotenone/antimycin A to inhibit mitochondrial complexes (Sigma-Aldrich), and 50 mm 2-deoxy-D-glucose inhibit glycolysis (Sigma-Aldrich). The metabolic profiles, including oxygen consumption rate (OCR) and extracellular acidification rate (ECAR), were measured using a Seahorse XF96 analyzer (Agilent technologies). Data were normalized to protein content for analysis.

### Detection of NAD^+^ and NAD^+^/NADH

Primary hepatocytes were lysed, and NAD^+^ and NADH levels were measured using the NAD^+^/NADH assay kit (Abcam) according to the manufacturer's protocol. Briefly, cell lysates were centrifuged, and the supernatant was used for the assay, which quantifies NADH by a colorimetric reaction at 450 nm. NAD^+^ levels were determined using a standard curve. The NAD^+^/NADH ratio was calculated and normalized to protein content.

### ATP and ATP/AMP measurement

ATP and AMP levels were measured using the ATP/AMP assay kit (Abcam). Briefly, cells were lysed in lysis buffer, and the lysates were centrifuged at 12,000 × g for 10 min at 4 °C to remove debris. The supernatant was then used for ATP and AMP quantification. ATP and AMP levels were measured by luminescent detection, following the manufacturer's instructions. Standard curves for ATP and AMP were generated for quantification. The ATP/AMP ratio was calculated, and values were normalized to total protein concentration.

### Isotope tracing analysis

To analyze glucose metabolism in mice, we utilized ^13^C-glucose isotope tracing. Mice were fasted overnight and then administered a bolus of [^U-13^C] glucose (500 mg/kg) via intraperitoneal injection. Blood and liver tissues were collected 2huors post-injection. Metabolites were extracted from the tissues and analyzed by liquid chromatography-mass spectrometry for isotopic enrichment and metabolic flux analysis. The incorporation of ^13^C-labeled carbon into various metabolites was quantified to assess glycolytic and oxidative pathways, including glucose utilization, lactate production, and TCA cycle intermediates.

### Metabolites measurement

Succinate and α-ketoglutarate concentrations in the liver tissues and serum were determined using a succinate colorimetric assay kit and α-ketoglutarate colorimetric assay kit (BioVision), according to the manufacturer's instructions.

### SDH activity assessment

The SDH activity in primary hepatocytes was measured according to the manufacturer's instructions using the succinate dehydrogenase activity colorimetric assay kit (BioVision).

### Cell cycle analysis

Cells were harvested, fixed in 70% ethanol, and stored at -20 °C overnight. After fixation, cells were washed with PBS and incubated with propidium iodide (Beyotime Biotechnology) solution containing RNase A for 30 min at 37 °C in the dark. Flow cytometric analysis was performed using flow cytometer. Cell cycle distribution (G0/G1, S, and G2/M phases) was analyzed using FlowJo software. The percentage of cells in each phase was determined based on DNA content.

### Immunoprecipitation of complex II

Whole-cell extracts were prepared by solubilizing cells in 1% dodecyl maltoside supplemented with protease and phosphatase inhibitors (Thermo Fisher Scientific) on ice for 30 min. Immunoprecipitation of Complex II was performed using the Pierce Cross-link Immunoprecipitation kit (Thermo Fisher Scientific), which conjugated 5 μg of SDHA antibody (Cell Signaling Technology) to protein A/G agarose resin. The complex was immunoprecipitated from whole-cell extracts overnight at 4 °C with gentle rocking. Complex II was eluted according to the Pierce Cross-link IP kit protocol and the pH was adjusted to 9.5 using Tris-HCl before SDS-PAGE analysis.

### Blue native-page

Blue native polyacrylamide gel electrophoresis (BN-PAGE) was performed to separate protein complexes based on their size and charge. Briefly, cell lysates were prepared using 1% digitonin (Sigma-Aldrich) in a buffer containing 50 mm Tris-HCl, pH 7.5, 150 mm NaCl, and protease inhibitors (Roche) on ice for 30 min. Protein concentrations were determined using the BCA assay (Thermo Fisher Scientific). Samples were mixed with 5x native page sample buffer (Invitrogen) and 0.05% Coomassie G-250 (Bio-Rad), and incubated for 5 min at room temperature. Equal amounts of protein (20—40 µg) were loaded onto 4-16% gradient native page gels (Invitrogen). Electrophoresis was carried out at 150 V in a cold room for 30 min. Protein transfer was performed using the Bio-Rad Transblot Turbo semi-dry transfer system at 15 V for 15 min in 2× nupage transfer buffer (Invitrogen) containing 10% methanol. Following transfer, proteins were fixed to the Immobilon-FL PVDF membrane (Millipore) with 8% acetic acid (Sigma-Aldrich) for 5min. Immunoblotting analysis was subsequently performed according to standard protocols. Quantification of IBs was performed using ImageJ.

### Mitochondrial calcium measurement

Mitochondrial calcium was measured using the FRET-based probe 4mtD3CPV (Addgene). This probe includes cyan fluorescent protein (CFP) as the donor and yellow fluorescent protein (YFP) as the acceptor. Calcium binding alters the FRET efficiency, enabling real-time monitoring of mitochondrial calcium levels. Cells were transfected with 4mtD3CPV using Lipofectamine 3000 (Invitrogen) and imaged every 10 s post-transfection using a confocal microscope, and 90 s later, 100 µM ATP was injected. CFP/YFP excitation was set at 430 nm, with emission at 475 nm and 535 nm, respectively. The FRET signal was quantified by the YFP/CFP intensity ratio. Furthermore, hepatocytes were incubated with Rhod2-AM (Yeasen Biotechnology), 5 µM for 30 min at 37°C, followed by washing and a 30 min incubation to DAPI. Mitochondrial calcium was imaged using a confocal microscope.

### ER calcium measurement

ER calcium levels were measured using the genetically encoded calcium indicator G-CEPIA1er (Addgene). This probe exhibits fluorescence changes in response to calcium binding within the ER. Hepatocytes were transfected with G-CEPIA1er using Lipofectamine 3000 (Invitrogen) according to the manufacturer's protocol. After 24 h, cells were imaged by confocal microscope. After 10sec of baseline recording, a single pulse of 100 µM ATP was delivered to liberate calcium stores and then washed out. The peak amplitudes of calcium responses to 100 µM ATP were normalized to the basal fluorescence (F₀) measured prior to stimulation. The area under the curve of the bar graph was calculated by multiplying the change in fluorescence relative to the baseline (ΔF/F₀) by the time (s).

### Analysis of the influx and efflux of ER calcium

For this experiment, Cells were permeabilized with 20 µM β-escin (Sigma-Aldrich) in intracellular medium. After washing with ICM for 5min, cells were superfused with loading buffer to load calcium stores. Then, release buffer with 1µM IP3 (Sigma-Aldrich) was applied to stimulate IP3R. The peak calcium response was normalized to basal fluorescence (F₀), and the ER Ca²⁺ release rate (ΔF/Fmax) was calculated over time (s).

### In situ proximity ligation assessment

Protein-protein interactions were detected using duolink in situ detection kit (Sigma-Aldrich) by following the manufacturer's instructions. Hepatocytes were fixed with 4% paraformaldehyde, and permeabilized by 0.2% triton x. Then, incubated with blocking solution, followed by overnight incubation with primary antibodies. The cells were then incubated with secondary antibodies (Invitrogen) conjugated to oligonucleotides, followed by ligation and amplification. The fluorescence signals were analyzed by confocal microscope. Primary antibodies used in in situ PLA experiments were VDAC1 (Cell Signaling Technology), GRP75 (Cell Signaling Technology), IP3R1(Cell Signaling Technology).

### Transmission electron microscopy

Transmission electron microscopy of hepatocytes was performed according to the manufacturer's protocol. The sections were further incubated with 0.3% lead citrate and imaged using an electron microscope (HITACHI).

### Immunoprecipitation coupled with mass spectrometry

Briefly, the cells were transfected with the target plasmid and lysed in ice-cold IP buffer (25 mM HEPES, pH 7.4, 150 mM NaCl, 1 mM EDTA, 1% NP-40, protease inhibitors). Immunoprecipitation was performed using primary antibodies and protein A/G-agarose beads (Santa Cruz Biotechnology). The beads were washed, and the immunoprecipitated complexes were eluted in SDS sample buffer and separated by SDS-PAGE. Proteins were visualized by silver staining (Thermo Fisher Scientific), and specific bands were excised, digested with trypsin, and analyzed by LC-MS (Thermo Fisher Scientific).

### Co-immunoprecipitation assay

The desired plasmid was successfully transfected into the cells, which were then lysed using ice-cold IP buffer. The cell lysates were incubated overnight at 4 °C with the specified antibody against the tag, followed by precipitation with protein A/G-agarose beads (Santa Cruz Biotechnology). After washing the beads, the protein complexes were resuspended in SDS sample buffer and analyzed by western blotting.

### Docking analysis

Molecular docking of Parkin (2ZEQ) and Sigma-1 (AF-O55242-F1) was carried out using Autodock. The protein structures were obtained from UniProt, and both were prepared by removing water molecules and heteroatoms, followed by energy minimization. Docking simulations were performed to predict the binding affinity and interaction mode. The top docking poses were analyzed to identify key binding residues, and the results were visualized using Pymol.

### Ubiquitination detection

Cells were transfected with the target plasmid, and 10 µM MG132 (Sigma-Aldrich) was added to the culture medium for 6 h. The cells were then harvested, and the lysates were collected. To prepare the samples, the lysates were diluted 10-fold with IP lysis buffer (Thermo Fisher Scientific), followed by ultrasound disruption and centrifugation according to the manufacturer's instructions. Ubiquitination levels were assessed by western blotting.

### Statistical analyses

Data are expressed as mean ± SEM. Statistical comparisons between two groups were performed using unpaired student's t-test. For multiple comparisons, one-way or two-way ANOVA accompanied by Bonferroni post hoc tests, as appropriate. A p-value of <0.05 was considered statistically significant. All statistical analyses were conducted using GraphPad Prism 8.

## Results

### Hepatocyte specific PINK1 deletion impairs liver regeneration

**Figure 1:**
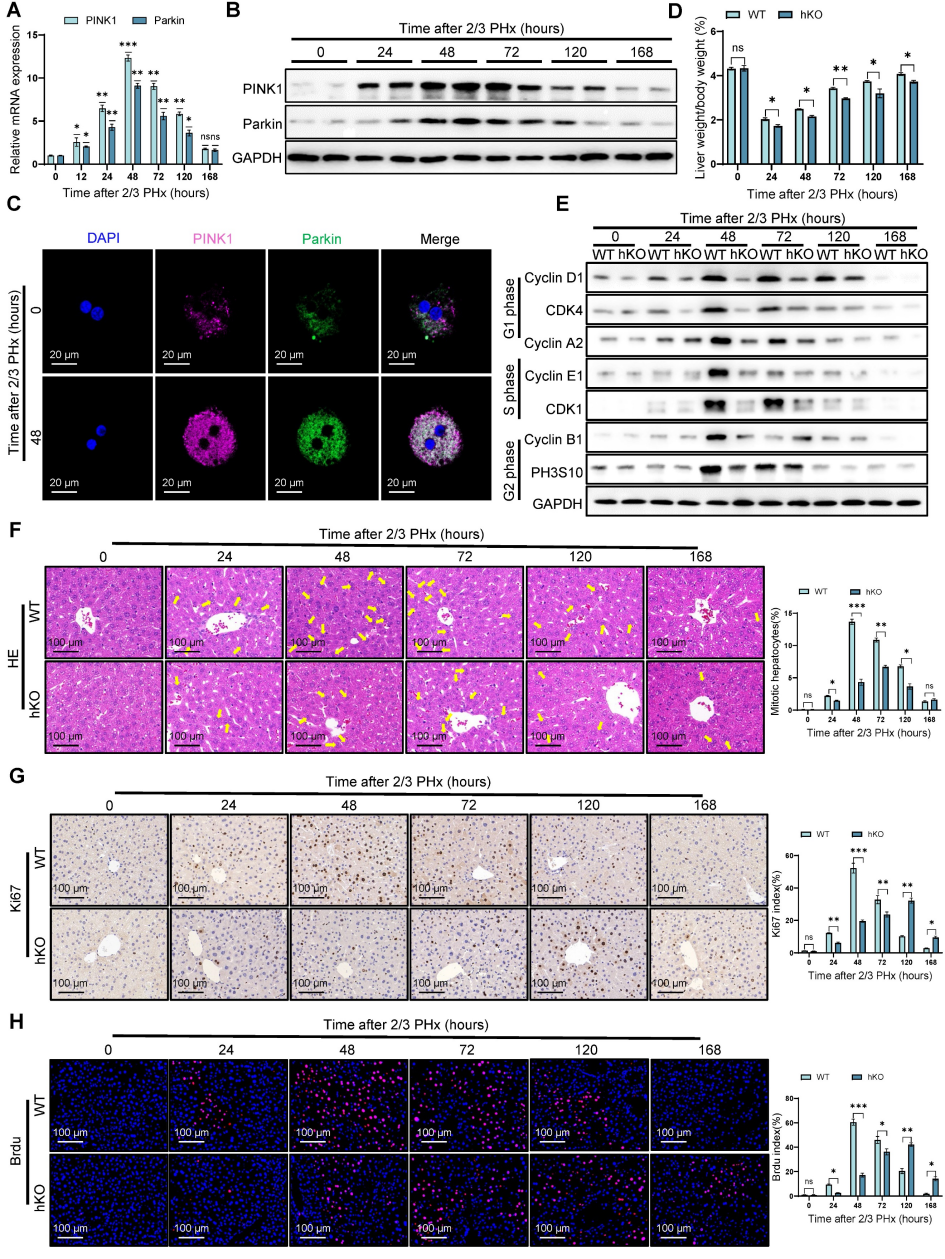
**PINK1 depletion impairs liver regeneration.** (A) The relative mRNA expression of PINK1, Parkin in liver tissues at different time after PHx; (B) PINK1, Parkin protein expression levels in liver tissues at different time after PHx; (C) Immunofluorescence staining of PINK1, Parkin in primary hepatocytes isolated from livers after PHx; (D) The ratios of liver weight/body weight at different time points after PHx; (E) Protein expression of cell cycle markers at various times following PHx; (F) HE staining of liver tissues at different time after PHx; (G) Immunohistochemistry of Ki67 in liver tissues at different time after PHx; (H) Immunofluorescence staining of Brdu in liver tissues at different time post PHx. Data were presented as mean ± SEM; n = 4-6 per group; *P < 0.05, **P < 0.01, ***P < 0.001.

To investigate PINK1 expression dynamics during liver regeneration, liver samples were collected from mice at multiple time points following PHx. PINK1/Parkin mRNA levels increased significantly, peaking at 48 h post hepatectomy, then gradually returning to baseline (Figure 1A). These results were confirmed by western blotting and immunofluorescence (Figure 1B, C) (Figure S1A), suggesting a potential role for PINK1 in liver regeneration.

To assess the functional contribution of PINK1, hepatocyte specific PINK1 deficiency mice (hKO) was induced using the Alb-Cre/loxP system (Figure S1B, C). Following PHx, serum levels of alanine aminotransferase (ALT), aspartate aminotransferase (AST), and bilirubin remained significantly elevated in mice with PINK1 deficiency compared to wild-type (WT) mice (Figure S1D), indicating delayed hepatic functional recovery. At the cellular level, cyclins associated with cell proliferation were downregulated in livers from mice with PINK1 deficiency (Figure 1E). The mitotic index was lower in these mice compared to WT mice (Figure 1F). Immunohistochemical staining for Ki67 and bromodeoxyuridine (BrdU) revealed significantly fewer proliferating cells and a slower rate of proliferation in the livers of mice with PINK1 deficiency post PHx (Figure 1G, H). In summary, hepatocyte specific PINK1 depletion impairs liver regeneration through disruption of cell cycle progression and reduced hepatocyte proliferation.

### PINK1 hepatocyte deficiency leads to mitochondrial dysfunction during liver regeneration

**Figure 2:**
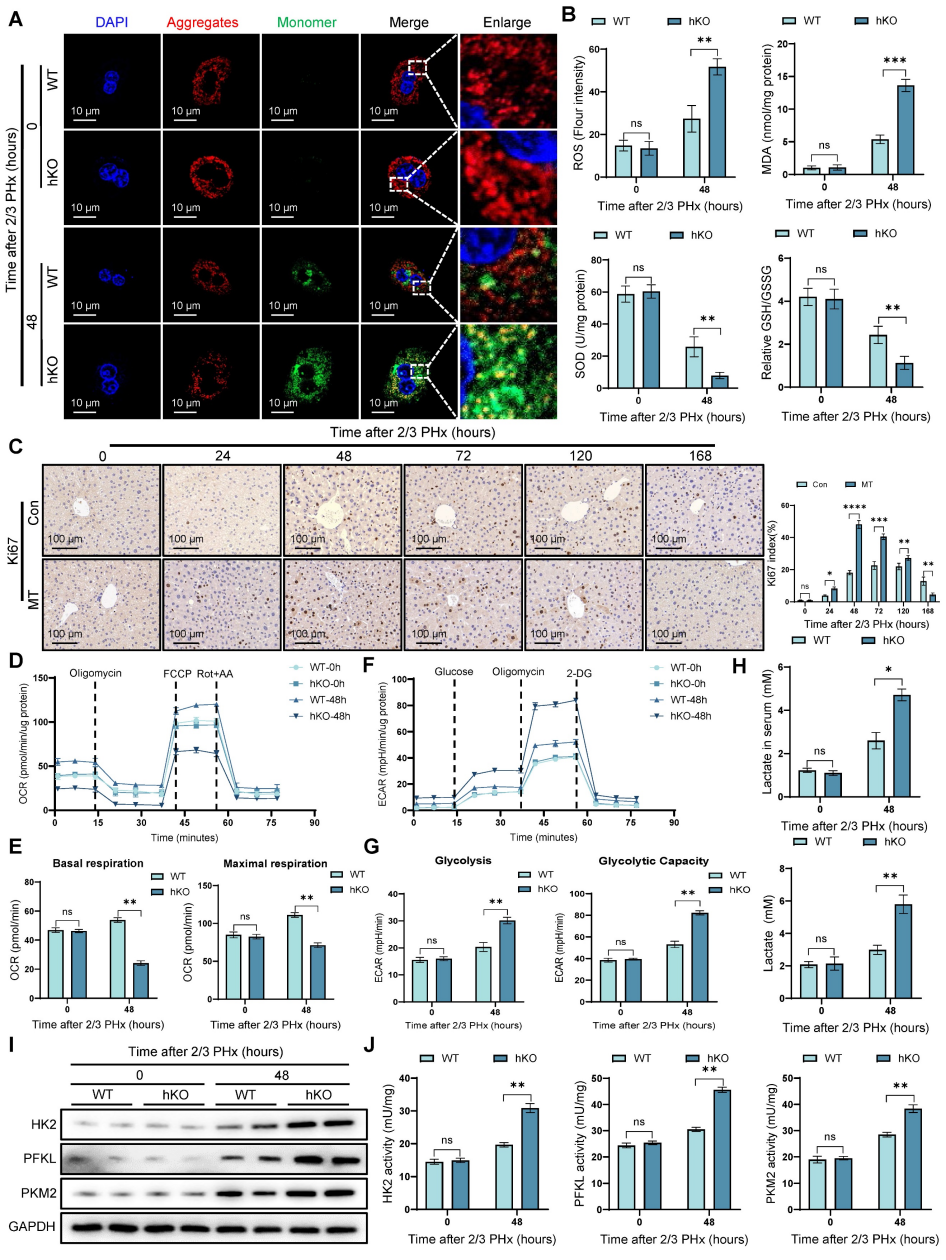
** PINK1 deletion leads to mitochondrial dysfunction in hepatocytes during liver regeneration.** (A) Mitochondrial membrane potential of primary hepatocytes isolated from livers post PHx were measured using JC10 dye, aggregates (Red), monomer (Green); (B) ROS, MDA, SOD and GSH/GSSG levels in liver tissues at different time after PHx; (C) Immunohistochemistry of Ki67 in liver tissues at different time after PHx with or without MitoTEMPO pre-treatment; (D) Primary hepatocytes OCR was measured after PHx by XF-analyzer; (E) Quantification of OCR; (F) Primary hepatocytes ECAR was measured after PHx by XF-analyzer; (G) Quantification of ECAR; (H) Hepatocytes and serum lactate assay after PHx; (I) HK2, PFKL and PKM2 protein expression in hepatocytes after PHx; (J) HK2, PFKL and PKM2 activity detection in hepatocytes after PHx. Data were presented as mean ± SEM; n = 4-6 per group; *P < 0.05, **P < 0.01, ***P < 0.001.

Liver regeneration requires substantial energy supply, and mitochondria, as the cell's energy hub, play a critical role in liver regeneration 25. JC-10 staining revealed a more significant decrease in mitochondrial membrane potential in PINK1 deficient cells (Figure 2A) (Figure S2A). At the same time, oxidative stress measurements showed an increase in oxidative stress levels in PINK1absent hepatocytes (Figure 2B), which was further confirmed by tissue DHE staining (Figure S2B, C). Next, we used MitoTEMPO, a specific mitochondrial ROS scavenger, to deplete mitochondrial ROS. After treatment, we observed an elevated expression of cyclin proteins (Figure S2D), along with an increase in Ki67 positive cells in liver tissue (Figure 2C). We then used seahorse analysis to examine mitochondrial respiration parameters in isolated primary hepatocytes. The results showed a reduction in both basal respiration and maximal oxygen consumption rate in PINK1 deficient hepatocytes (Figure 2D, E), while extracellular acidification rate was increased (Figure 2F, G). Meanwhile, we found that NAD^+^ and NAD^+^/NADH ratio decreased in hepatocytes, indicating impaired mitochondrial function and glycolytic activity is enhanced (Figure S2E). Furthermore, the detection of lactate in serum and liver cells revealed that the lactate level increased during the liver regeneration process in hKO mice (Figure 2H). Moreover, the expression and activity of HK2, PFKL, and PKM2 are significantly elevated in hepatocytes (Figure 2I, J). Our data demonstrate that PINK1 knockout hepatocytes exhibit mitochondrial dysfunction during liver regeneration, characterized by impaired mitochondrial respiration and a compensatory increase in glycolysis.

### Deletion of PINK1 disrupts the TCA cycle in regenerating hepatocytes and leads to succinate accumulation

**Figure 3:**
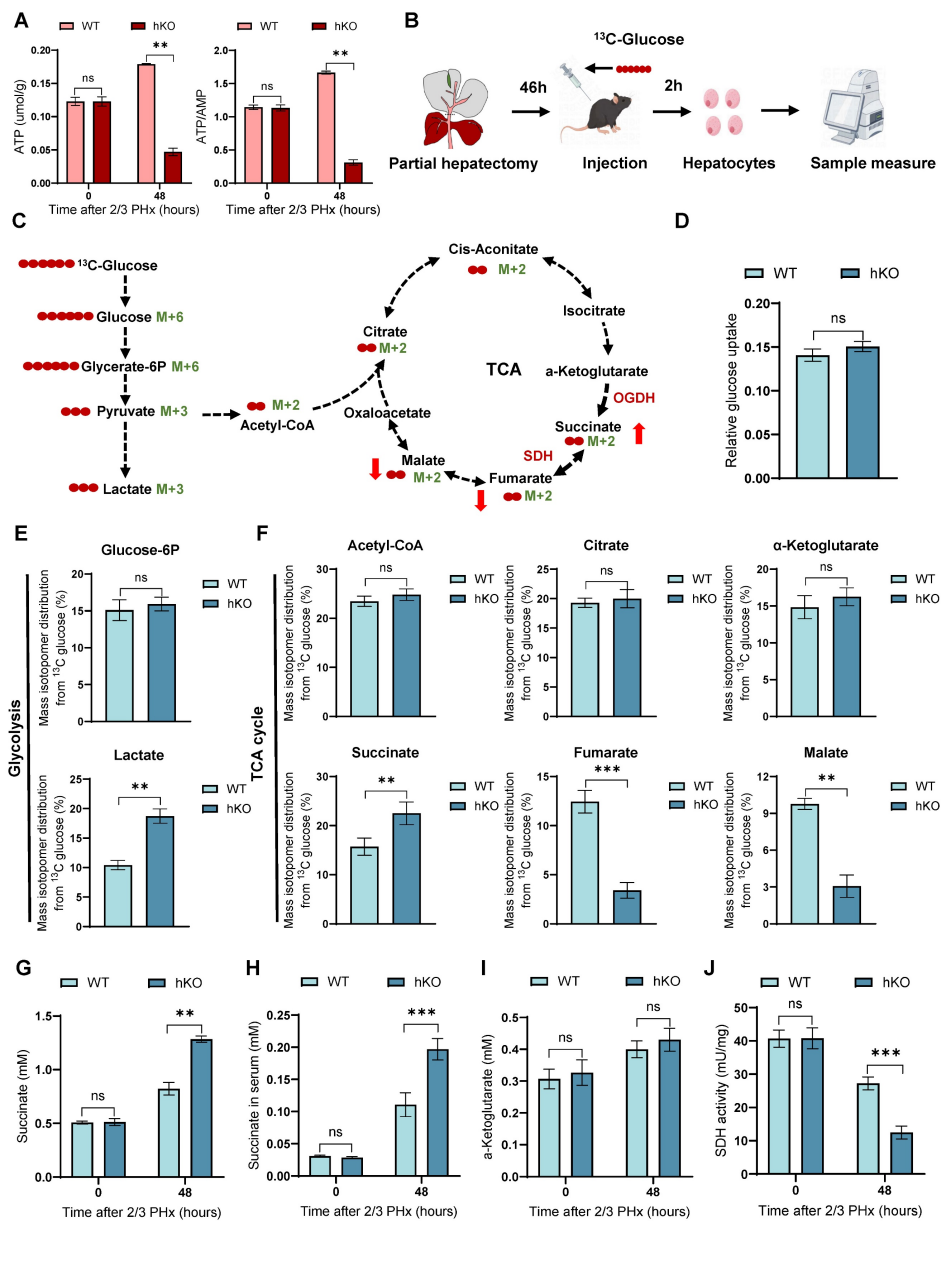
** Loss of PINK1 in hepatocytes disrupts the TCA cycle and leads to succinate accumulation during liver regeneration.** (A) ATP and ATP/AMP levels in primary hepatocytes post PHx; (B) Experimental setup for 13C-glucose labeling. Mice were injected with [U-^13^C6] glucose, and primary hepatocytes were harvested at specific time points for metabolic analysis; (C) Schematic diagram of glucose metabolism tracing in hepatocytes. Red circles represent carbons derived from [U-^13^C6] glucose; (D) Glucose uptake wase determined in hepatocytes isolated from livers post PHx; (E) Mass isotopologue distributions of glucose in glycolysis including glucose-6p and lactate in hepatocytes isolated from livers post PHx; (F) Mass isotopologue distributions of glucose in TCA cycle metabolites including acetyl-coa, citrate, α-ketoglutarate, succinate, fumarate and malate in hepatocytes post PHx; (G) Succinate levels in liver tissues post PHx; (H) Succinate levels in serum post PHx; (I) α-ketoglutarate levels in liver tissues post PHx; (I) SDH activity in primary hepatocytes post PHx. Data were presented as mean ± SEM; n = 4-6 per group; *P < 0.05, **P < 0.01, ***P < 0.001.

TCA cycle serves as the primary hub for cellular energy production 10. In previous studies, we observed that PINK1 deletion resulted in glucose metabolic reprogramming in regenerating hepatocytes, characterized by a reduction in mitochondrial oxygen consumption and an increase in extracellular acidification. We next evaluated ATP production in hepatocytes during regeneration and detected a pronounced decline in hKO mice (Figure 3A), indicating impaired energy metabolism. To elucidate the role of PINK1 in regulating hepatocyte metabolism during liver regeneration, we employed 13C-glucose tracers to assess glucose metabolism in hepatocytes 48 h post PHx (Figure 3B, C). Glucose consumption rates remained comparable between hKO and WT mice (Figure 3D). However, we observed a significant alteration in the glucose conversion profile. Specifically, PINK1 ablation led to enhanced glycolytic activity, as evidenced by an increase in lactate levels, while glucose-6-phosphate levels remained unchanged (Figure 3E). Notably, PINK1 deletion impaired the TCA cycle in hepatocytes, resulting in accumulated succinate and diminished fumarate and malate, without affecting acetyl-CoA, citrate, or α-ketoglutarate levels. (Figure 3F). This change was further corroborated by increased succinate levels in both liver tissues and serum (Figure 3G, H).

To investigate the underlying cause of succinate accumulation in the absence of PINK1, we further measured α-ketoglutarate levels and found no significant differences between hKO and WT mice (Figure 3I). Moreover, the activity of α-ketoglutarate dehydrogenase (OGDH) showed no significant changes (Figure S3A). Succinate dehydrogenase (SDH), which catalyzes the conversion of succinate to fumarate, plays a crucial role in the TCA cycle, serves as both a key metabolic regulator and a reliable indicator of TCA cycle activity 12, 26. We next assessed SDH activity and found that PINK1 deletion significantly reduced SDH activity in hepatocytes following liver resection (Figure 3J). Importantly, when primary hepatocytes were treated with exogenous succinate, no changes in SDH activity were observed, ruling out any potential negative feedback regulation due to succinate accumulation (Figure S3B). In summary, these findings support the conclusion that PINK1 deletion impairs SDH activity in regenerating hepatocytes, thereby disrupting the TCA cycle and contributing to succinate accumulation during liver regeneration.

### Hepatocyte succinate paracrine regulation of macrophages inhibits liver regeneration

**Figure 4:**
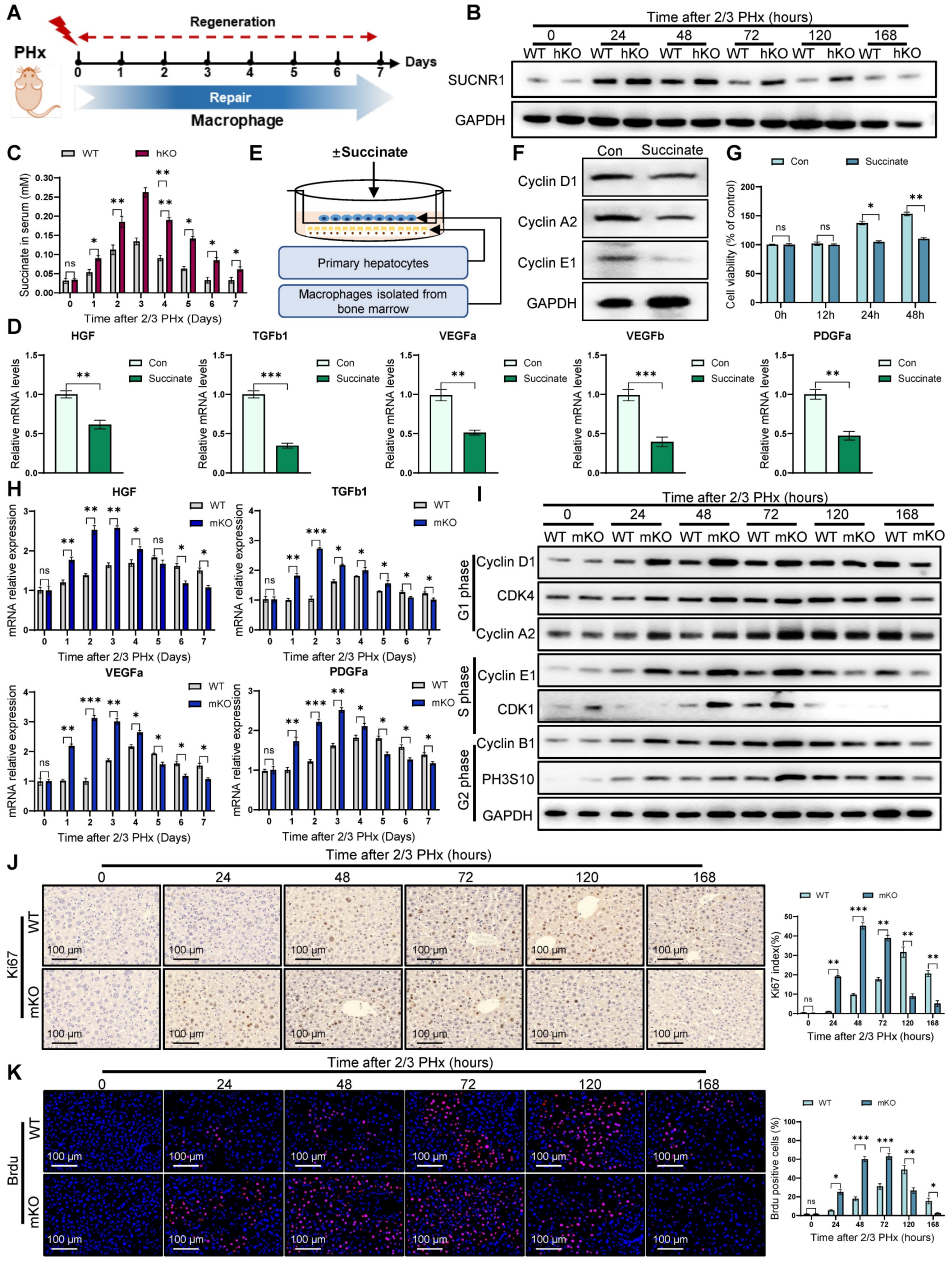
** Hepatocyte-derived succinate regulates macrophages to inhibit liver regeneration.** (A) Schematic illustration showing the role of macrophages in tissue repair after PHx; (B) Protein expression of SUCNR1 in macrophages isolated from livers at different time post PHx; (C) Succinate levels in serum at different time post PHx; (D) The relative mRNA expression of repair-related genes in BMDMs treated with or without succinate; (E) Schematic drawing showing that primary hepatocytes co-cultured with BMDMs pretreated with or without succinate; (F) Protein expression of cyclin D1, cyclin A2 and cyclin E1 in primary hepatocytes co-cultured with BMDMs; (G) The cell viability of primary hepatocytes co-cultured with BMDMs were detected by CCK-8 assay; (H) The relative mRNA expression of repair-related genes in macrophages isolated from livers at different time post PHx in WT and mKO mice; (I) Protein expression of cell cycle markers at various times following PHx in WT and mKO mice; (J) Immunohistochemistry of Ki67 in liver tissues at different time after PHx in WT and mKO mice; (K) Immunofluorescence staining of Brdu in liver tissues at different time after PHx in WT and mKO mice. Data were presented as mean ± SEM; n = 4-6 per group; *P < 0.05, **P < 0.01, ***P < 0.001.

Macrophages are critical for tissue homeostasis and repair. Studies have demonstrated that liver macrophages produce IL-6, which promotes hepatic proliferation via the STAT3 signaling pathway 27. In addition, cytokines such as HGF and TGFβ, secreted by macrophages during regeneration, also modulate hepatocyte proliferation 28. These findings underscore the potential pro-regenerative role of macrophages in liver regeneration (Figure 4A). To investigate the role of macrophages in liver regeneration, we depleted macrophages using clodronate-liposomes (CL-lipos). As expected, CL-lipos treatment significantly impaired liver regeneration in WT mice, confirming the essential role of macrophages in hepatic repair. (Figure S4A).

Furthermore, we explored whether this accumulation of succinate in hepatocytes could paracrinally affect macrophages, thereby modulating liver regeneration. Western blotting revealed that following liver resection, the succinate receptor SUCNR1 of macrophages was progressively upregulated in hKO mice (Figure 4B). Consistently, serum succinate levels were significantly elevated at multiple time (Figure 4C). To further investigate the impact of succinate on macrophages, we performed in vitro experiments by extracting bone marrow-derived macrophages (BMDMs) and stimulating them with exogenous succinate. The results showed that after succinate stimulation, the expression of repair-related genes in macrophages, such as HGF, TGFβ1, VEGFa, VEGFb, and PDGFα, was downregulated (Figure 4D). In vivo, we observed similar downregulation of these repair factors in liver macrophages of hKO mice (Figure S4B). Additionally, a co-culture system was established using succinate-treated macrophages and the primary hepatocytes (Figure 4E). WB and CCK8 assays confirmed that macrophages treated with succinate inhibited the proliferation of hepatocytes (Figure 4F, G). Meanwhile, flow cytometry and EdU analysis demonstrated that succinate-pretreated macrophages inhibited the cell cycle progression of AML cells (Figure S4C-E).

To further assess the role of succinate and macrophages in liver regeneration, we generated a bone marrow-specific SUCNR1 knockout mouse (mKO) using the Lyz2-Cre/loxp system. We found that macrophage SUCNR1 knockout increased repair related cytokines expression post PHx (Figure 4H). Moreover, cell cycle analysis demonstrated that liver regeneration occurred more rapidly in mKO mice (Figure 4I). Histological analysis of Ki67, BrdU, and PCNA staining further confirmed that myeloid-specific knockout of SUCNR1 accelerated liver regeneration following PHx (Figure 4J, K) (Figure S4F). In conclusion, our results shown that hepatocyte-derived succinate in hKO mice inhibits macrophage pro-reparative functions through the SUCNR1 receptor, thereby affecting liver regeneration.

### Hepatocyte PINK1 ablation leads to mitochondrial calcium overload and inhibition of SDH activity

**Figure 5:**
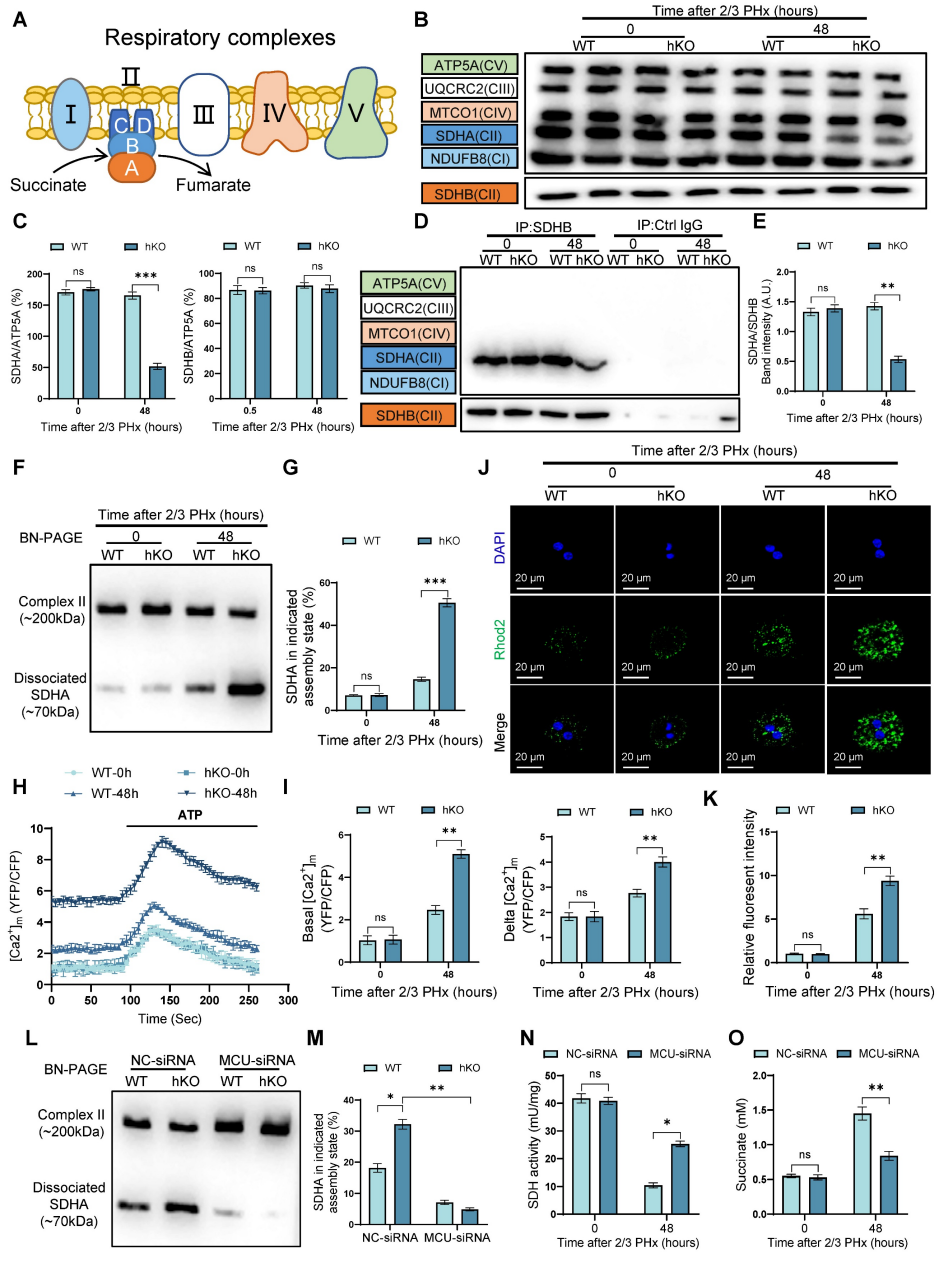
** PINK1 deletion in hepatocytes causes mitochondrial calcium overload and impairment of SDH activity.** (A) Schematic illustration of the five major respiratory complexes; (B) Native IBs were performed to assess assembly of CI-V in primary hepatocytes post PHx; (C) Quantification of SDHA and SDHB subunit levels normalized to ATP5A signal; (D) Immunoprecipitated assays were conducted to investigate the interaction between SDHB and CI-V in primary hepatocytes post PHx; (E) Quantification of immunoprecipitated SDHA normalized to SDHB levels; (F) BN-PAGE and SDHA immunoblot analysis of primary hepatocytes post PHx. SDHA blot intensity profile revealing Complex II-associated SDHA (~200 kDa) and dissociated SDHA (~70 kDa); (G) Proportion of SDHA in complex II versus dissociated SDHA populations; (H) Mitochondrial calcium detection by the FRET based 4mtD3CPV in primary hepatocytes post PHx; (I) Quantitative analysis of basal and ATP-stimulated mitochondrial calcium accumulation; (J) Detection of mitochondrial calcium in primary hepatocytes after PHx by rhod-2. (K) Quantification of mean fluorescence intensity; (L) BN-PAGE and SDHA immunoblot analysis of primary hepatocytes from WT and hKO mice transfected with MCU-siRNA or NC-siRNA post PHx. SDHA blot intensity profile revealing Complex II-associated SDHA (~200 kDa) and dissociated SDHA (~70 kDa); (I) Proportion of SDHA in complex II versus dissociated SDHA populations; (N) SDH activity of hepatocytes from hKO mice transfected with MCU-siRNA or NC-siRNA post PHx; (O) Succinate level of hepatocytes from hKO mice transfected with MCU-siRNA or NC-siRNA post PHx. Data were presented as mean ± SEM; n = 4-6 per group; *P < 0.05, **P < 0.01, ***P < 0.001.

SDH is also Complex II of the respiratory chain, consisting of four subunits (SDHA, SDHB, SDHC, and SDHD) encoded by nuclear DNA (Figure 5A). To investigate how PINK1 deficiency in hepatocytes impacts SDH activity during liver regeneration, we hypothesized that PINK1 deficiency might influence the abundance or assembly of respiratory complex II in regenerating hepatocytes. Immunoblot analysis revealed that PINK1 deletion specifically resulted in the selective reduction of SDHA subunit, whereas SDHB remained unaffected (Figure 5B, C). Notably, subunits of other complexes were not significantly altered (Figure 5B). Furthermore, we performed co-immunoprecipitation experiments using SDHB to assess the integrity of complex II assembly. As expected, the interaction between the SDHA subunit and SDHB decreased (Figure 5D, E). Validation of this result by BN-PAGE further revealed a reduction of complex II, alongside an increase in the dissociated SDHA form (Figure 5F, G). These findings suggest that PINK1 ablation disrupts complex II stability, thereby impairing SDH enzymatic activity.

Given the established role of mitochondrial calcium influx in promoting the dissociation of complex II 29, we hypothesized that altered calcium signaling could contribute to the observed SDH dissociation in PINK1 deficiency hepatocytes. Using a fluorescent resonance energy transfer (FRET)-based mitochondrial calcium probe (4mtD3CPV), we detected a significant increase in mitochondrial calcium levels in PINK1 ablation hepatocytes both at baseline and following ATP stimulation after PHx (Figure 5H, I). These findings were further corroborated using mitochondrial calcium ion fluorescent probes (Figure 5J, K), confirming that PINK1 ablation leads to mitochondrial calcium overload. Interestingly, using MCU-siRNA to inhibit mitochondrial calcium uptake, we found that MCU knockdown reversed the dissociation of the SDHA subunit (Figure 5L, M). Moreover, this intervention led to elevated SDH enzymatic activity and concomitant reduction of intracellular succinate accumulation (Figure 5N, O). In conclusion, our results demonstrate that PINK1 knockout in hepatocytes during liver regeneration induces mitochondrial calcium overload, which, in turn, triggers the dissociation of SDH subunits and inhibits its enzymatic activity.

### PINK1 inhibits ER-mitochondria calcium transport to promote liver regeneration

**Figure 6:**
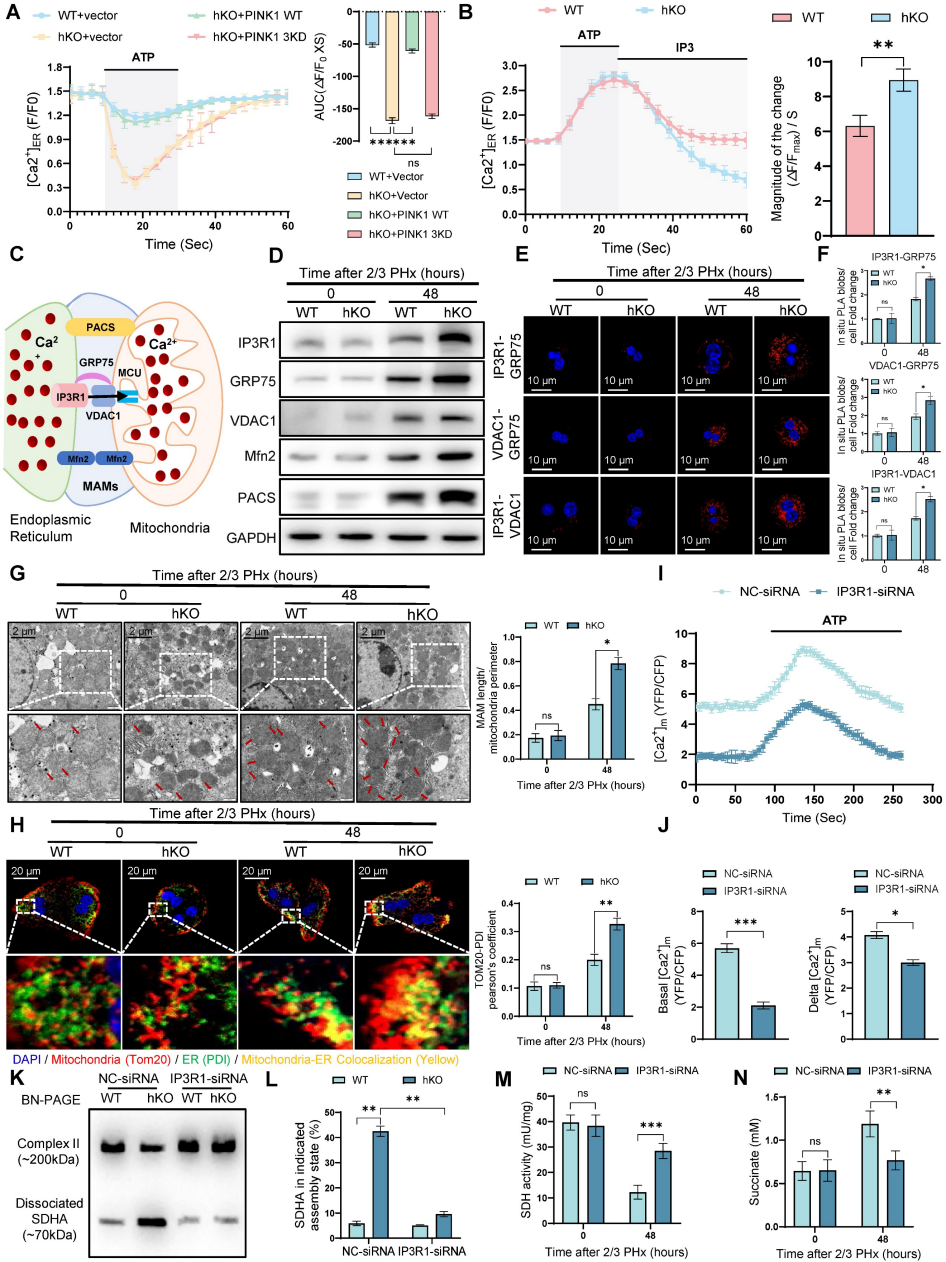
** PINK1 suppresses ER-mitochondria calcium transport to enhance liver regeneration.** (A) ER calcium measurement by G-CEPIA1er in primary hepatocytes after PHx from WT and hKO mice transfected with PINK1 WT, 3KD mutant, or empty vector; (B) Measurement of ER calcium uptake and release in primary hepatocytes after PHx; (C) Schematic illustration of MAMs and calcium transport; (D) Protein expression of MCC complex and PACS, Mfn2 in primary hepatocytes after PHx; (E) Analysis of interaction between MCC complex proteins in primary hepatocytes after PHx by in situ PLA; (F) Quantification of IP3R1-GRP75, VDAC1-GRP75, IP3R1-VDAC1 interaction; (G) TEM examine the physical interaction between mitochondria and the ER in primary hepatocytes after PHx; (H) Immunofluorescence staining of TOM20 (red) and PDI (green) in primary hepatocytes after PHx; (I) Mitochondrial calcium detection in primary hepatocytes after PHx from hKO mice transfected with IP3R-siRNA or NC-siRNA by FRET-based 4mtD3CPV; (J) Quantitative analysis of basal and ATP-stimulated mitochondrial calcium accumulation; (K) BN-PAGE and SDHA immunoblot analysis of primary hepatocytes after PHx from WT and hKO mice transfected with IP3R-siRNA or NC-siRNA. SDHA blot intensity profile revealing Complex II-associated SDHA (~200 kDa) and dissociated SDHA (~70 kDa); (L) Proportion of SDHA in complex II versus dissociated SDHA populations; (M) SDH activity of primary hepatocytes from hKO mice transfected with IP3R-siRNA or NC-siRNA after PHx. (N) Succinate level of hepatocytes from hKO mice transfected with IP3R-siRNA or NC-siRNA post PHx. Data were presented as mean ± SEM; n = 4-6 per group; *P < 0.05, **P < 0.01, ***P < 0.001.

The ER serves as a critical cellular calcium reservoir, playing a pivotal role in maintaining calcium homeostasis within both the cytoplasm and mitochondria. To investigate the involvement of the ER in regulating mitochondrial calcium overload in hepatocytes following PHx in hKO mice, we treated primary hepatocytes from both hKO and WT mice 48 h post PHx with ATP. ATP induces the production of inositol 1,4,5-trisphosphate (IP3), which binds to the IP3R to facilitate calcium release from the ER 30. Using the ER-specific calcium indicator G-CEPIA1er, we observed that ATP treatment of hepatocytes from hKO mice resulted in an enhanced calcium release from the ER (Figure 6A). Notably, this increase in calcium release was suppressed by exogenous expression of wild-type PINK1 (PINK1 WT), but not by the kinase-dead mutant (PINK1 3KD) (Figure 6A). To explore the mechanism by which PINK1 regulates ER calcium release, we first evaluated the activity of ER calcium channels. The sarco/endoplasmic reticulum Ca^2+^-ATPase (SERCA) pumps calcium from the cytosol into the ER, while IP3R mediates calcium release in response to IP3. Using G-CEPIA1er, we assessed the impact of PINK1 loss on both calcium uptake and release in the ER. After permeabilizing cells with β-escin, we stimulated calcium influx through SERCA using calcium chloride, followed by IP3 treatment to trigger calcium release via IP3R. Interestingly, while PINK1 deficiency did not affect calcium uptake into the ER, it resulted in a significant increase in calcium release (Figure 6B).

The ER and mitochondria form dynamic microdomains, known as mitochondria-associated membranes (MAMs), which are maintained by specialized tethering proteins that mediate inter-organelle communication 31. MAMs facilitate the transfer of calcium, phospholipids, and metabolites between the ER and mitochondria. Calcium transfer at MAMs is regulated by the formation of the MAM calcium channel (MCC) complex (Figure 6C). Western blot analysis revealed that 48 h after PHx, the expression of MCC complex proteins was elevated in PINK1 knockout hepatocytes, along with an upregulation of MAM-associated proteins PACS and Mfn2 (Figure 6D). To further assess the formation of the MCC complex, we performed in situ PLA, which detect protein proximity within 40 nm. We found that PINK1 deletion significantly increased the proximity of MCC complex proteins in hepatocytes after PHx (Figure 6E, F). TEM of primary hepatocytes from WT and hKO mice post PHx revealed a notable increase in the proportion of mitochondria in close proximity to the ER (Figure 6G). Immunostaining of ER and mitochondria with PDI and TOM20, respectively, followed by confocal microscopy, demonstrated extensive co-localization of the ER and mitochondria in PINK1-deficient hepatocytes after PHx (Figure 6H). These findings suggest that PINK1 deficiency promotes the formation of MAMs and MCC complexes during liver regeneration.

To further validate these observations, we used IP3R-siRNA to inhibit MCC complex formation in hKO mice, thereby blocking calcium transport between the ER and mitochondria. IP3R inhibition significantly reduced mitochondrial calcium levels in PINK1 deficiency hepatocytes after PHx (Figure 6I, J). BN-PAGE and immunoblot analysis demonstrated that IP3R inhibition reversed the dissociation of SDH (Figure 6K, L). Furthermore, we observed increased SDH activity and elevated succinate levels in liver tissue (Figure 6M, N). Seahorse analysis revealed that in vivo inhibition of IP3R led to increased oxygen consumption rate (Figure S5A, B) and enhanced ATP production in liver cells (Figure S5C). Functionally, inhibiting MCC complex formation mitigated the impairment of liver regeneration, as evidenced by BrdU staining (Figure S5D). In summary, these results indicate that PINK1 deletion in hepatocytes enhances calcium transfer from the ER to mitochondria at MAMs, leading to the dissociation of SDH and impaired enzymatic activity, which ultimately inhibits liver regeneration.

### Parkin promotes the ubiquitin-mediated degradation of sigma-1, thereby inhibiting IP3R-mediated calcium transport in MAMs

**Figure 7:**
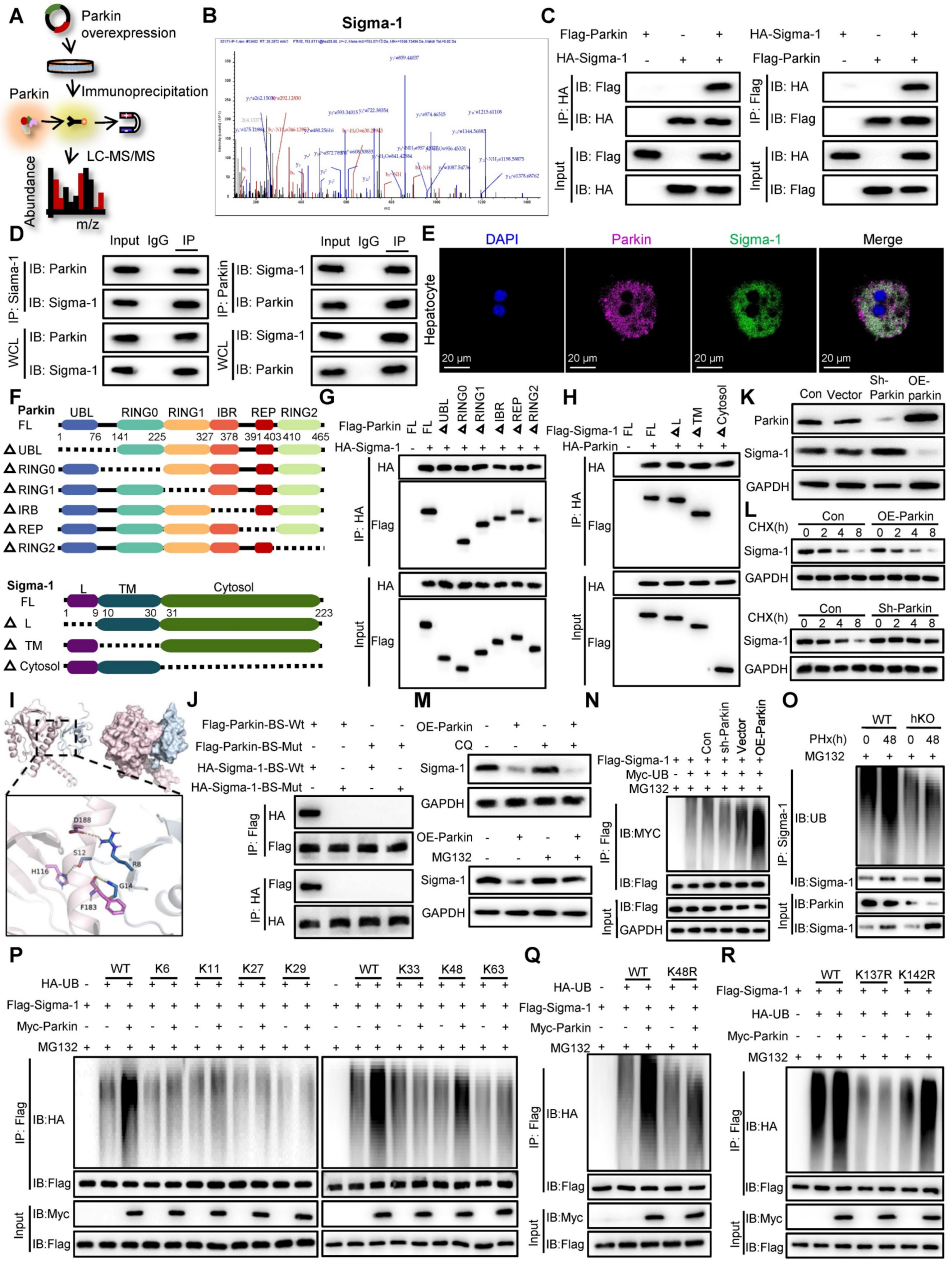
** Parkin promotes ubiquitin-mediated degradation of Sigma-1 and inhibits IP3R-mediated calcium transport in MAMs.** (A) Schematic illustration of IP-MS analysis to identify the target of Parkin; (B) The peptide sequences of the Sigma-1 protein identified in the Co-IP complex through mass spectrometry; (C) Co-IP assays were performed to examine the interaction between exogenous Parkin and Sigma-1 in HEK293T cells; (D) Co-IP assays were performed to examine the interaction between endogenous Parkin and Sigma-1 in hepatocytes; (E) Immunofluorescence staining of Parkin, Sigma-1 in primary hepatocytes post PHx; (F) Schematic diagram depicting the structural domains of Parkin and its truncated mutants and Sigma-1 and its truncated mutants; (G,H) Co-IP assays were performed to investigate the binding regions of Parkin and Sigma-1; (I) The simulated 3D triad structures of Parkin and Sigma-1; (J) IP analysis showing the interaction of Flag- Parkin -BS-Wt or Flag- Parkin -BS-Mut with HA- Sigma-1-BS-Wt or HA- Sigma-1-BS-Mut in HEK293T cells; (K) Expression levels of Sigma-1 in AML cells with Parkin knockdown or overexpression; (L) Expression levels of Sigma-1 in AML cells with Parkin knockdown or overexpression were treated with CHX for the indicated times; (M) The protein expression levels of Sigma-1 in AML cells with Parkin overexpression were measured following treatment with CQ or MG132; (N) Ubiquitination levels of Sigma-1 in AML cells after Parkin knockdown or overexpression; (O) Ubiquitination levels of Sigma-1 in primary hepatocytes post PHx; (P) Ubiquitination screening of Sigma-1 by Parkin with various types of ubiquitin (K6, K11, K27, K29, K33, K48, and K63); (Q) Ubiquitination levels of Sigma-1 in HEK293T cells transfected with Myc-Parkin, Flag-Sigma-1, and HA-UB or its K48R mutant; (R) Ubiquitination levels of Sigma-1 in HEK293T cells transfected with Myc-Parkin, Flag-Sigma-1 or its K137R and K142R mutants, and HA-UB. Data were presented as mean ± SEM; n = 4-6 per group; *P < 0.05, **P < 0.01, ***P < 0.001.

To explore the potential mechanisms by which PINK1/Parkin signaling modulates calcium transfer in MAMs, we conducted IP-MS to identify proteins that interact with Parkin (Figure 7A). After screening for MAM-associated proteins with high interaction scores, Sigma-1 emerged as a candidate target that interacts with and is regulated by Parkin (Figure 7B). Sigma-1 functions as a molecular chaperone for IP3R, maintaining its structural integrity 23. To confirm this interaction, we performed co-immunoprecipitation assays, both exogenously and endogenously, which validated a direct interaction between Parkin and Sigma-1 (Figure 7C, D). Furthermore, confocal microscopy of primary hepatocytes 48 h after partial PHx revealed colocalization of Parkin and Sigma-1 (Figure 7E). To delineate the specific regions involved in the interaction between Parkin and Sigma-1, we generated a series of mutants (Figure 7F). The results revealed that the UBL domain of Parkin (residues 1-76) and the cytosolic region of Sigma-1 (residues 31-223) are essential for their interaction (Figure 7G, H). Additionally, 3D structural simulations and docking studies indicated that amino acids R8, S12, and G14 of Parkin interact with residues D188, H116, and F183 of Sigma-1 (Figure 7I). Mutation of these binding sites to alanine abolished the interaction between Parkin and Sigma-1, as demonstrated by Co-IP (Figure 7J).

Given that Parkin is an E3 ubiquitin ligase known to mediate the ubiquitin-proteasome degradation of its substrates, we next investigated whether Parkin influences the expression levels of Sigma-1. Western blot analysis showed that knockdown of Parkin resulted in increased Sigma-1 expression, whereas overexpression of Parkin led to a decrease in Sigma-1 levels (Figure 7K). Moreover, in the presence of the protein synthesis inhibitor cycloheximide (CHX), Parkin clearly decreased the half-life of Sigma-1 (Figure 7L). The proteasome inhibitor MG132, but not chloroquine, reversed the downregulation of Sigma-1 induced by Parkin overexpression, suggesting that Parkin regulates Sigma-1 stability through the proteasomal degradation pathway (Figure 7M).

We further examined the role of Parkin in the ubiquitination of Sigma-1. In vitro ubiquitination assays confirmed that Parkin upregulation enhanced, while Parkin downregulation suppressed, the ubiquitination of Sigma-1 (Figure 7N). Consistently, we confirmed in vivo analysis (Figure 7O). Moreover, Parkin was found to specifically promote K48-linked ubiquitination of Sigma-1 (Figure 7P). The K48R mutant of ubiquitin failed to mediate Sigma-1 ubiquitination, reinforcing the importance of K48 linkage (Figure 7Q). Finally, to identify the specific ubiquitination site on Sigma-1, we generated Sigma-1 mutants and found that Parkin could not mediate K48-linked ubiquitination at lysine 137 (K137R) (Figure 7R). These results indicate that Parkin specifically promotes K48-linked ubiquitination of Sigma-1 at the K137 site.

### Sigma-1 is required for PINK1/Parkin in liver regeneration

**Figure 8:**
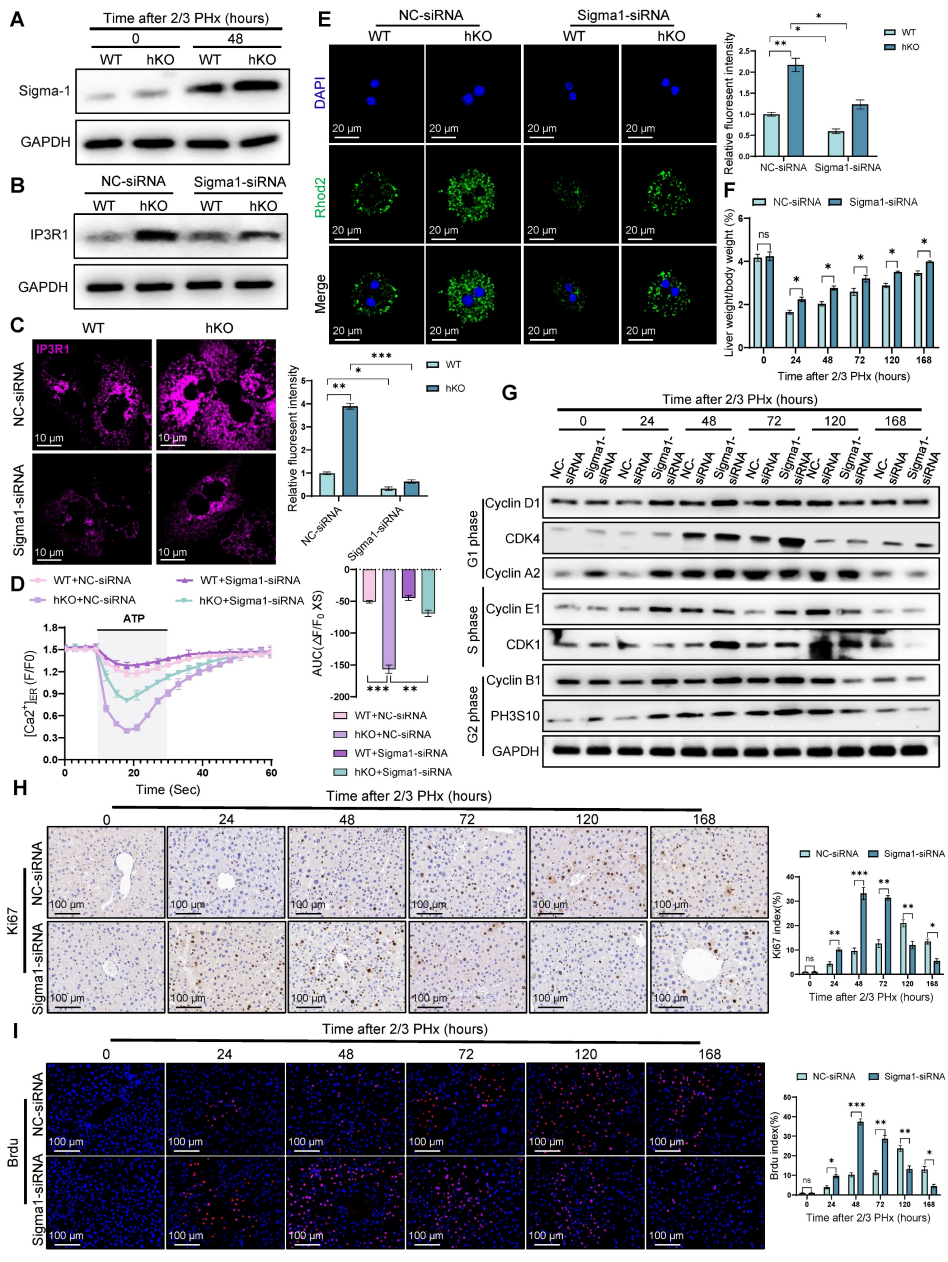
** Sigma-1 is indispensable for PINK1/Parkin dependent liver regeneration.** (A) Protein expression of Sigma-1 in primary hepatocytes from WT and hKO mice after PHx; (B) Protein expression of IP3R1 in primary hepatocytes from WT and hKO mice transfected with Sigma1-siRNA or NC-siRNA after PHx; (C) Immunofluorescence staining of IP3R1 in primary hepatocytes from WT and hKO mice transfected with Sigma1-siRNA or NC-siRNA after PHx; (D) ER calcium measurement by G-CEPIA1er in primary hepatocytes after PHx from WT and hKO mice transfected with Sigma1-siRNA or NC-siRNA; (E) Detection of mitochondrial calcium in primary hepatocytes from WT and hKO mice transfected with Sigma1-siRNA or NC-siRNA after PHx by rhod-2. (F) The ratios of liver weight/body weight at different time points after PHx; (G) Protein expression of cell cycle markers at various times following PHx; (H) Immunohistochemistry of Ki67 in liver tissues at different time after PHx; (I) Immunofluorescence staining of Brdu in liver tissues at different time post PHx. Data were presented as mean ± SEM; n = 4-6 per group; *P < 0.05, **P < 0.01, ***P < 0.001.

Notably, Sigma-1 expression was significantly upregulated in regenerating hepatocytes of hKO mice compared to WT mice (Figure 8A), suggesting its potential regulatory role in this process. To determine the necessity of Sigma-1's functional requirement in mediating MAM-dependent calcium transfer between ER and mitochondria in regenerating hepatocytes, we performed rescue experiments using Sigma-1 specific siRNA to achieve targeted knockdown. Western blot and immunofluorescence staining results indicated that Sigma-1 knockdown significantly attenuated IP3R1 expression in hepatocytes during liver regeneration (Figure 8B, C). Furthermore, we observed that inhibiting Sigma-1 can attenuated the release of calcium from ER in hKO mice hepatocytes (Figure 8D). Meanwhile, fluorometric analysis using mitochondrial-targeted calcium indicators demonstrated significantly reduced calcium in hepatocytes following Sigma-1 inhibition compared to controls (Figure 8E), indicating its regulatory function in calcium flux across MAMs interfaces. To further validate the direct role of Sigma-1 in liver regeneration in hKO mice, we found that inhibiting the expression of Sigma-1 could efficiently restore the impaired liver regeneration caused by PINK1 deficiency in hepatocytes (Figure 8F-I). Taken together, these results suggested that Sigma-1 in hepatocytes of hKO mice affects IP3R1-mediated calcium transfer at MAMs, thereby inhibiting liver regeneration.

### Hepatocyte PINK1 overexpression enhances liver regeneration

**Figure 9:**
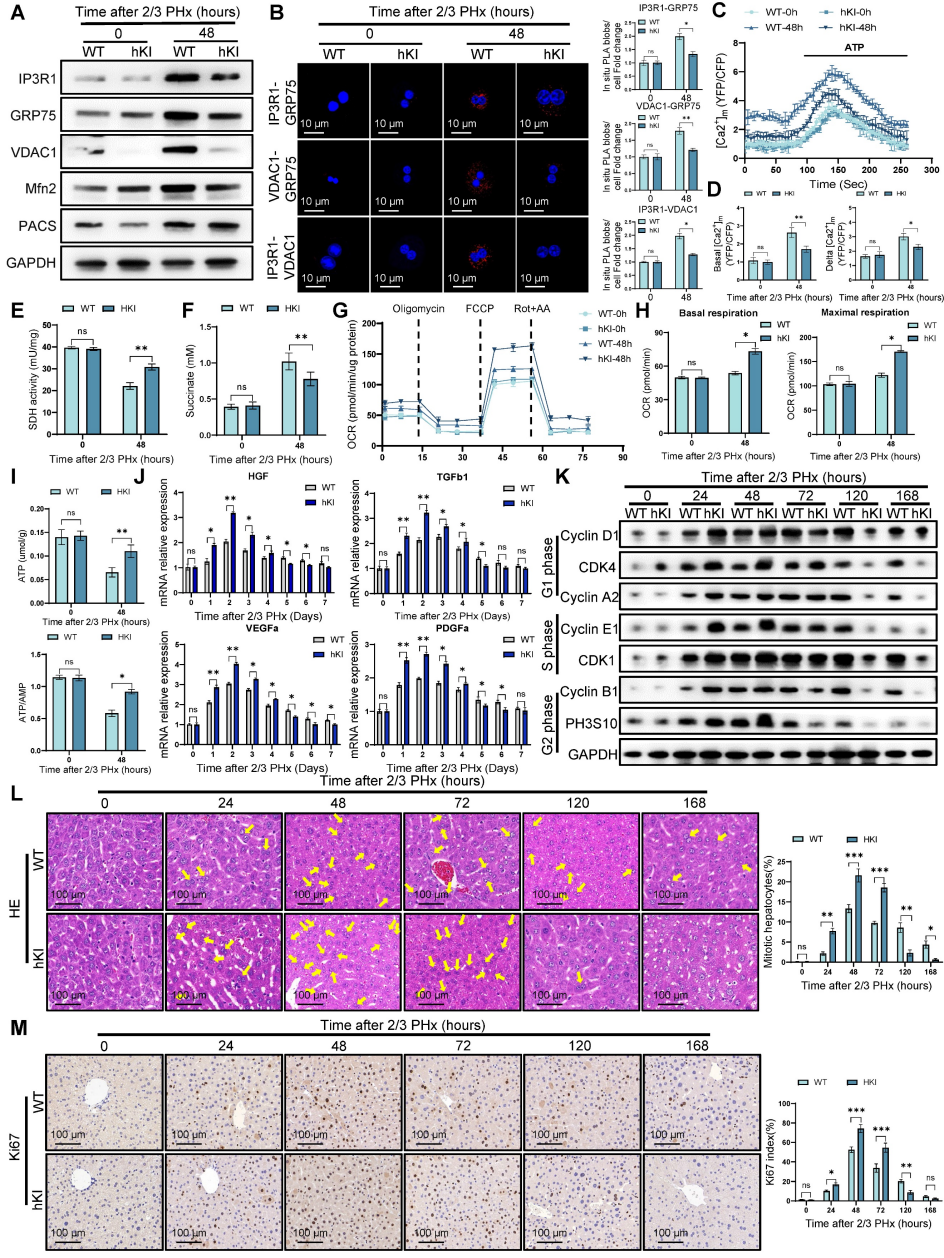
** Hepatocyte PINK1 overexpression promotes liver regeneration.** (A) Protein expression of MCC complex and PACS, Mfn2 in primary hepatocytes isolated from WT and hKI mice after PHx; (B) Analysis of interaction between MCC complex proteins in WT and hKI mice primary hepatocytes after PHx by in situ PLA and quantification of IP3R1-GRP75, VDAC1-GRP75, IP3R1-VDAC1 interaction; (C) Mitochondrial calcium detection by the FRET based 4mtD3CPV in primary hepatocytes from WT and hKI mice after PHx; (D) Quantitative analysis of basal and ATP-stimulated mitochondrial calcium accumulation; (E) SDH activity in primary hepatocytes from WT and hKI mice after PHx; (F) Succinate levels in WT and hKI mice liver tissues after PHx; (G) OCR of primary hepatocytes isolated from WT and hKI mice after PHx was measured by XF-analyzer; (H) Quantification of OCR; (I) ATP and ATP/AMP levels of primary hepatocytes from WT and hKI mice after PHx; (J) The relative mRNA expression of repair-related genes in macrophages isolated from WT and hKI mice after PHx; (K) Protein expression of cell cycle markers at various times following PHx; (L) HE staining of liver tissues at different time after PHx; (M) Immunohistochemistry of Ki67 in liver tissues at different time after PHx. Data were presented as mean ± SEM; n = 4-6 per group; *P < 0.05, **P < 0.01, ***P < 0.001.

To further investigate the role of PINK1 in liver regeneration, we employed CRISPR/Cas9 gene knock-in technology to generate hepatocyte-specific PINK1 overexpression (hKI) mice. As anticipated, PINK1 overexpression markedly inhibited the formation of MCC complexes and reduced the expression of MAMs related proteins, such as Mfn2 and PACS (Figure 9A). PLA analysis further demonstrated that PINK1 overexpression diminished the interaction between MCC complex proteins within hepatocytes following PHx (Figure 9B). Additionally, hKI mice exhibited significantly lower baseline mitochondrial calcium levels and attenuated ATP-stimulated calcium responses in hepatocytes compared to WT mice (Figure 9C, D). Moreover, hepatocytes from hKI mice showed enhanced SDH activity and a reduction in hepatic succinate levels (Figure 9E, F). Seahorse metabolic analysis revealed that, following PHx, PINK1 overexpression hepatocytes exhibited an increased oxygen consumption rate and elevated ATP production (Figure 9G-I). Analysis of liver macrophages isolated post PHx revealed a significant upregulation of macrophage repair related gene expression in hKI mice compared to WT mice (Figure 9J). Correspondingly, PINK1 overexpression in hepatocytes substantially promoted liver regeneration after PHx (Figure 9K-M). In summary, these findings underscore the pivotal role of PINK1 in liver regeneration, which operates by modulating hepatocyte TCA cycle activity and influencing the regulatory functions of liver macrophages during regeneration.

## Discussion

Adult liver tissue demonstrates a remarkable regenerative capacity in response to hepatic injury. During this process, a multitude of regeneration-promoting genes are activated, which drive compensatory proliferation of remaining hepatocytes 2, 32. Here, our study reveals that the expression of PINK1/Parkin in hepatocytes increases during liver regeneration following PHx. PINK1 knockout in mouse hepatocytes impairs liver regeneration, while transgenic overexpression of PINK1 enhances this process. Moreover, we observed that PINK1 deficiency hepatocytes during regeneration exhibited mitochondrial dysfunction, characterized by reduced SDH activity, which led to impairment of TCA cycle and accumulation of succinate in hepatocytes. In vitro and in vivo experiments indicated that succinate, in a paracrine manner, acts on SUCNR1 in liver macrophages, inhibiting the expression of macrophage repair-associated genes during liver regeneration. Interestingly, macrophage-specific deletion of SUCNR1 reversed the regulatory effect of exogenous succinate on macrophages. Further experiments demonstrated that during regeneration, Parkin, as an E3 ubiquitin ligase, mediates the ubiquitination and degradation of the molecular chaperone Sigma-1 that interacts with IP3R. Additionally, PINK1 knockout hepatocytes facilitated the stabilization of MCC complexes in MAMs through the molecular chaperone function of Sigma-1, thereby enhancing calcium transport from the endoplasmic reticulum to mitochondria, ultimately leading to mitochondrial calcium overload. Interestingly, we also found that mitochondrial calcium overload promoted dissociation of SDHA from the complex II, resulting in a loss of its enzymatic activity. These findings unveil the mechanisms by which PINK1 regulates liver regeneration in hepatocytes and provide new therapeutic targets for clinical recovery.

Mitochondria are essential for the proper functioning of organs, responsible for multiple critical metabolic processes. Among these, the TCA cycle and oxidative phosphorylation meet the energetic demands of regenerative processes, and promoting mitochondrial health during regeneration is crucial for tissue homeostasis. Previous studies have shown that zinc finger and homeobox 2 (ZHX2), a mitochondrial function regulator in hepatocytes, suppresses the expression of electron transport chain genes and reduces mitochondrial biogenesis and oxidative phosphorylation, thus inhibiting regeneration after PHx and acute liver injury 25. Another study has highlighted the critical role of the mitochondrial deacetylase SIRT3 in liver regeneration, where SIRT3 deficiency in hepatocytes results in mitochondrial dysfunction and impaired hepatocyte replication 33. Our study reveals that PINK1 deficiency during hepatocyte regeneration induces mitochondrial dysfunction, characterized by TCA cycle impairment and reduced ATP production. Intriguingly, this metabolic perturbation triggers compensatory activation of glycolysis, which alleviates bioenergetic deficits and meets the heightened energy demands of proliferating hepatocytes. We also found that SDH activity was decreased in PINK1deplesion hepatocytes, with elevated succinate levels in both hepatocytes and serum during regeneration.

Macrophages are the most abundant immune cells in the liver and participate in regulating all stages of liver disease, from initial tissue injury to chronic inflammation, fibrosis, and repair. During repair, macrophages initially adopt a pro-inflammatory phenotype, rapidly responding to infection and injury. Once the danger subsides, they acquire an anti-inflammatory phenotype to promote the resolution of inflammation and tissue repair, making macrophages key players in liver regeneration 34, 35. It is well established that the production and secretion of repair factors are critical for macrophage phenotypic shift during regeneration. However, the molecular mechanisms driving and maintaining the repair phenotype of macrophages remain unclear. Previous studies have shown that peroxisome proliferator-activated receptor gamma (PPARγ) plays an important role in macrophage differentiation and function. The deletion of macrophage PPARγ results in a significant delay in skeletal muscle regeneration 36. Recent studies suggest that reduced phosphorylation of PPARγ at threonine 166 in macrophages within injured tissues leads to increased lipid synthesis, further triggering STAT3-mediated growth factor expression 37. As mentioned earlier, we observed that PINK1 knockout in hepatocytes during regeneration led to increased succinate levels in both hepatocytes and serum, along with elevated expression of the succinate receptor SUCNR1 in macrophages. Given that succinate is a hydrophilic compound incapable of traversing lipid membranes, its extracellular effects must be mediated by cell-surface receptors such as SUCNR1. Recent studies have demonstrated that extracellular vesicles derived from tumor cells contain high levels of succinate, which can induce M1 polarization upon being phagocytized by macrophages, thereby promoting anti-tumor immune responses 38. However, the role of succinate in regulating macrophages via SUCNR1 during liver regeneration remains unclear. Previous studies have reported that exogenous succinate pre-treatment in BMDMs enhances LPS-induced expression of pro-inflammatory IL-1β while inhibiting the anti-inflammatory IL-10 expression 39. Another study demonstrated that knockout of uncoupling protein 1 (UCP1) in adipocytes decreased their ability to clear circulating succinate, leading to elevated extracellular succinate levels in liver tissue and driving inflammation through SUCNR1 in macrophages 40. Our study confirms that PINK1 deficiency in hepatocytes post PHx leads to succinate secretion, which, in a paracrine manner, acts on liver macrophages via SUCNR1, inhibiting the expression of macrophage repair-associated genes. Interestingly, depletion of SUCNR1 specifically in macrophages reversed the inhibitory effect of exogenous succinate on the repair phenotype.

MAMs are dynamic membrane structures between mitochondria and the endoplasmic reticulum, typically spanning distances of 10-50 nm, and serve as hotspots for the regulation of mitochondrial dynamics, autophagy, and redox homeostasis through various signaling pathways 41, 42. One of the key functions of MAMs is to regulate calcium transfer from the ER to mitochondria via the MCC complex, which is crucial for mitochondrial function 41. Dysregulated calcium transport within MAMs can disturb mitochondrial homeostasis and lead to mitochondrial dysfunction. Previous studies have reported that in alcoholic liver disease, PDK4 phosphorylation of its downstream target GRP75 promotes MCC complex formation, leading to mitochondrial calcium accumulation and dysfunction 43. Our results confirm that PINK1 deficiency in hepatocytes during regeneration increases the formation of MAMs and MCC complexes, promoting calcium transport from the ER to mitochondria, which causes mitochondrial calcium overload. However, the role of mitochondrial calcium overload in liver regeneration and its impact on the TCA cycle and related mechanisms remain unclear. It has been reported that mitochondrial calcium accumulation promotes the dissociation of complex II in the electron transport chain, leading to decreased SDH activity 29. Interestingly, we found that in PINK1 ablation hepatocytes after PHx, there was an increased dissociation of SDHA from the complex, then degradation by lysosomal. Further studies revealed that inhibiting MCU and IP3R expression in the MCC complex could suppress the dissociation of SDH in PINK1deficency hepatocytes, reverse TCA cycle dysfunction, and promote liver regeneration.

Sigma-1 is an ER protein specifically localized within MAMs, where it functions as a molecular chaperone for IP3Rs, maintaining their correct conformation 23. As a calcium transporter channel within MAMs, IP3R undergoes conformational changes and subsequent proteasomal degradation following calcium efflux. However, upon binding to IP3R, Sigma-1 enhances IP3R stability and facilitates calcium transport from the ER to mitochondria 23, 44. Previous studies have shown that the Sigma-1 agonist PRE-084 preserves calcium transfer and mitochondrial respiration, thereby alleviating the symptoms of Wolfram syndrome caused by WSF1 gene mutations 45. Additionally, a recent study reported that the small molecule Sigma-1 inhibitor CGI1746 suppresses iron-dependent cell death by inhibiting calcium transport from the ER to mitochondria 46. Intriguingly, we observed upregulated Sigma-1 expression in hepatocytes during liver regeneration in hKO mice. Inhibition of Sigma-1 significantly suppressed IP3R1 expression, suggesting a potential regulatory role of the Sigma-1/IP3R1 axis in liver regeneration. Parkin as an E3 ubiquitin ligase, is capable of recognizing and mediating the ubiquitination and degradation of ER or mitochondria-associated proteins within MAMs. Prior studies have suggested that Parkin specifically recognizes CISD1, an active regulator of IP3R, and mediates its ubiquitination, thereby inhibiting ER calcium release and maintaining cytosolic calcium homeostasis, which stabilizes Parkinson's disease-related phenotypes 47. However, it remains unclear whether Parkin interacts with Sigma-1 and plays a role in liver regeneration. Our study reveals that, during liver regeneration, Parkin recognizes Sigma-1 and exerts its E3 ligase activity, mediating Sigma-1's ubiquitination and degradation, thereby maintaining mitochondrial calcium homeostasis. To further validate the direct role of Sigma-1 in liver regeneration, we found that inhibiting Sigma-1 expression partially restored the impaired liver regeneration caused by PINK1 deficiency in hepatocytes. These findings underscore the role of Sigma-1 in modulating the stability of IP3R1 during liver regeneration.

In summary, we demonstrate the critical role of the PINK1/Parkin pathway in liver regeneration. Hepatocyte specific PINK1 knockout impairs liver regeneration following PHx, while PINK1 overexpression promotes liver regeneration. The loss of PINK1 in hepatocytes increased calcium transport within MAMs, resulting in mitochondrial TCA cycle dysfunction and a reduction in the expression of macrophage-mediated repair genes, ultimately inhibiting liver regeneration.

## Supplementary Material

Supplementary figures.

## Abbreviations

ALT: alanine aminotransferase
AST: aminotransferase
Brdu: bromodeoxyuridine
BMDMs: bone marrow-derived macrophages
ER: endoplasmic reticulum
ECAR: extracellular acidification rate
ERAD: ER-associated protein degradation
GRP75: glucose-regulated protein 75
HGF: hepatocyte growth factor
IP3: inositol 1,4,5-trisphosphate
LAMP1: lysosome-associated membrane protein 1
MAMs: mitochondria-associated ER membrane
MCC: MAM calcium channel
OCR: oxygen consumption rate
OGDH: α-ketoglutarate dehydrogenase
OXPHOS: oxidative phosphorylation
PHx: partial hepatectomy
PINK1: PTEN induced kinase 1 Gene
SDH: succinate dehydrogenase
TCA: tricarboxylic acid cycle
