# Supplementary material for: PINK1/Parkin promotes liver regeneration via Sigma-1 ubiquitination to inhibit ER-Mitochondrial calcium transfer
Source: Theranostics. 2026 Jan 1;16(3):1410–31. doi: 10.7150/thno.115726 (PMC12679573; doi:10.7150/thno.115726)
Supplement: Supplementary file 1 — Supplementary figures. [file thnov16p1410s1.pdf]

PINK1/Parkin promotes liver regeneration via Sigma-1 ubiquitination to inhibit ER-Mitochondrial calcium transfer

Jian Xu [abc#], Yuechen Wang[abc#], Weizhe Zhong[abc#], Haoran Hu[abc#], Ye Zhang[abc], Yiyun Gao[abc], Ping Wang[abc], Zhuqing Rao[d*], Haoming Zhou[abc*], Xuehao Wang[abc*]

Table of contents

Fig. S1 2

Fig. S2 3

Fig. S3 4

Fig. S4 5

Fig. S5 6

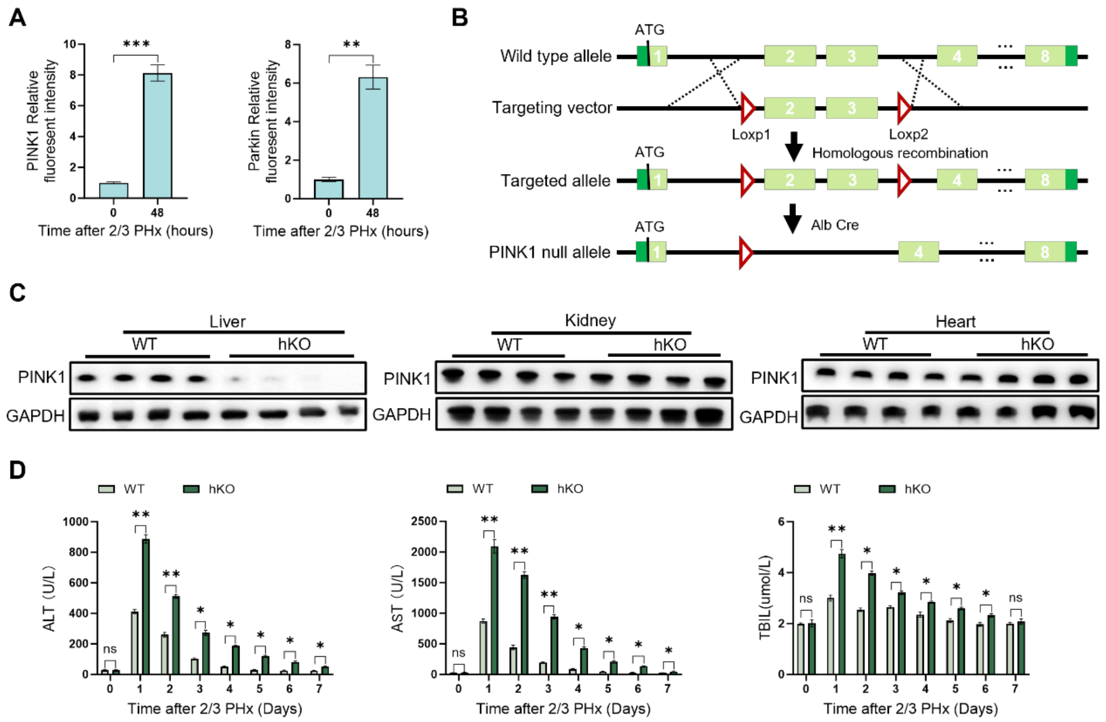

PINK1 deficient impairs liver regeneration. (A) Qualification of immunofluorescence staining of PINK1 and Parkin in primary hepatocytes after PHx; (B) Schematic diagram of the construction of hepatocyte-specific PINK1 knockout mice; (C) Western blot analysis of PINK1 in liver, kidney and heart tissues; (D) Serum ALT, AST and TBIL levels of WT and hKO mice after PHx. Data were presented as mean±SEM; n=4–6 per group; *$P < 0.05$, **$P < 0.01$, ***$P < 0.001$.

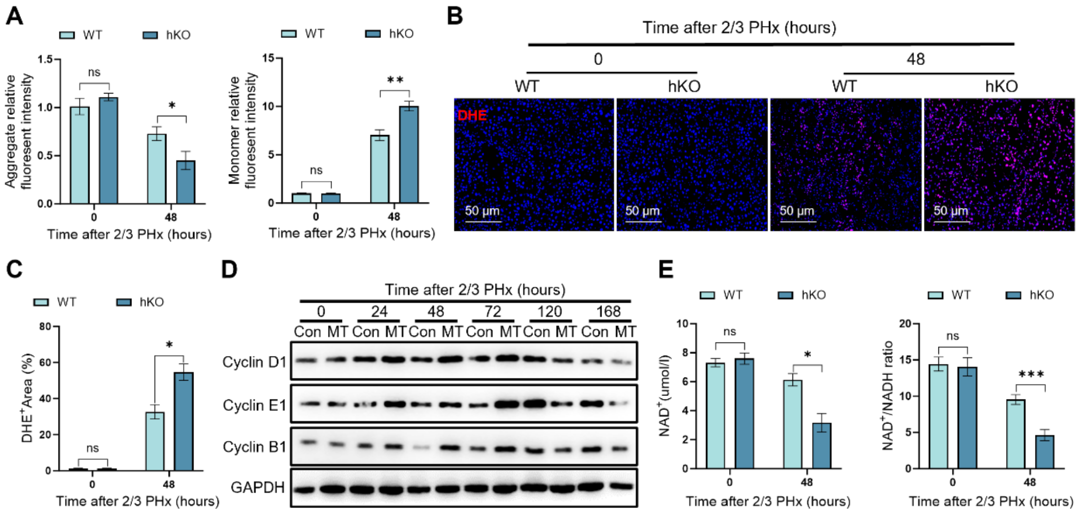

PINK1 deficiency causes mitochondrial dysfunction in hepatocytes during liver regeneration. (A) Qualification of mitochondrial membrane potential in primary hepatocytes after PHx; (B) DEH staining of liver tissues after PHx; (C) Qualification of immunofluorescence staining of DHE; (D) Protein expression of cell cycle markers at various time points after PHx in hKO mice treated with or without mito-tempo; (E) NAD^+ and NAD^+/NADH levels in liver tissues at different time after PHx. Data were presented as mean±SEM; n=4–6 per group; *P < 0.05, **P < 0.01, ***P < 0.001.

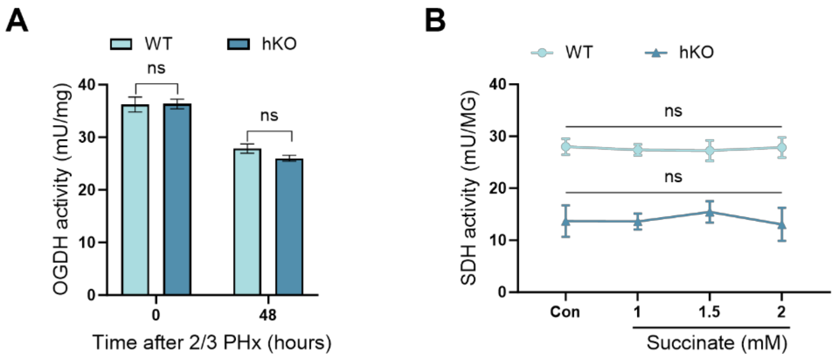

The absence of PINK1 in hepatocytes impairs the TCA cycle, causing an accumulation of succinate during liver regeneration. (A) OGDH activity detection in primary hepatocytes from WT and hKO mice after PHx; (B) SDH activity detection in primary hepatocytes stimulated by exogenous succinate with different concentration. Data were presented as mean±SEM; n=4–6 per group; *P < 0.05, **P < 0.01, ***P < 0.001.

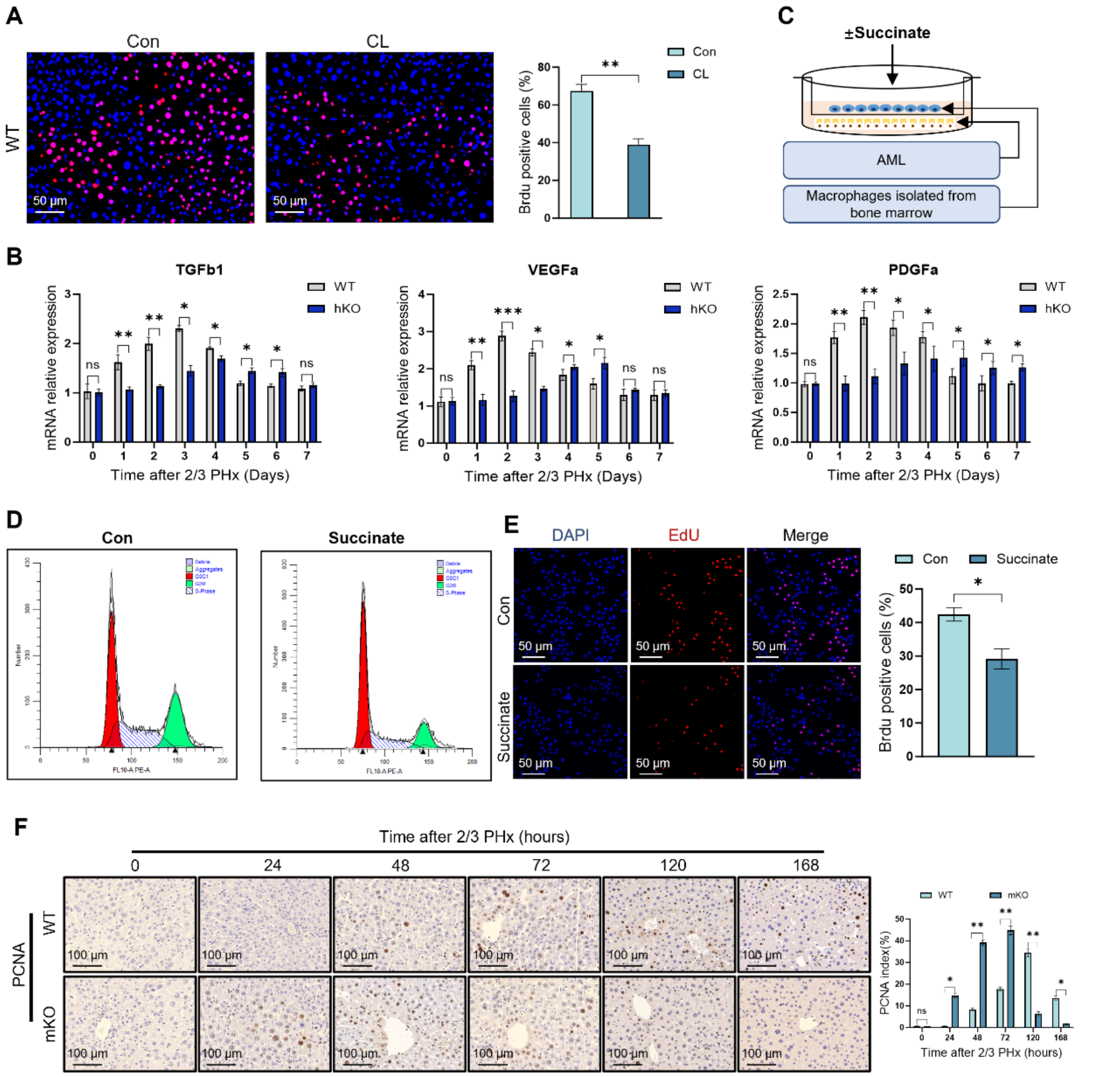

Myeloid-specific SUCNR1 knockout promotes liver regeneration. (A) Immunohistochemistry of Brdu in liver tissues 48h after PHx with or without CL pre-treatment; (B) The relative mRNA expression of repair-related genes in macrophages isolated from WT and hKO mice after PHx; (C) Schematic drawing showing that AML co-cultured with BMDMs pretreated with or without succinate; (D) Cell cycle analysis of AML co-cultured with BMDMs pretreated with or without succinate; (E) EdU incorporation reveals proliferative activity in AML co-cultured with BMDMs pretreated with or without succinate; (F) Immunohistochemistry of PCNA in liver tissues at different time after PHx in WT and mKO mice. Data were presented as mean±SEM; n=4–6 per group; $*P < 0.05$, $**P < 0.01$, $***P < 0.001$.

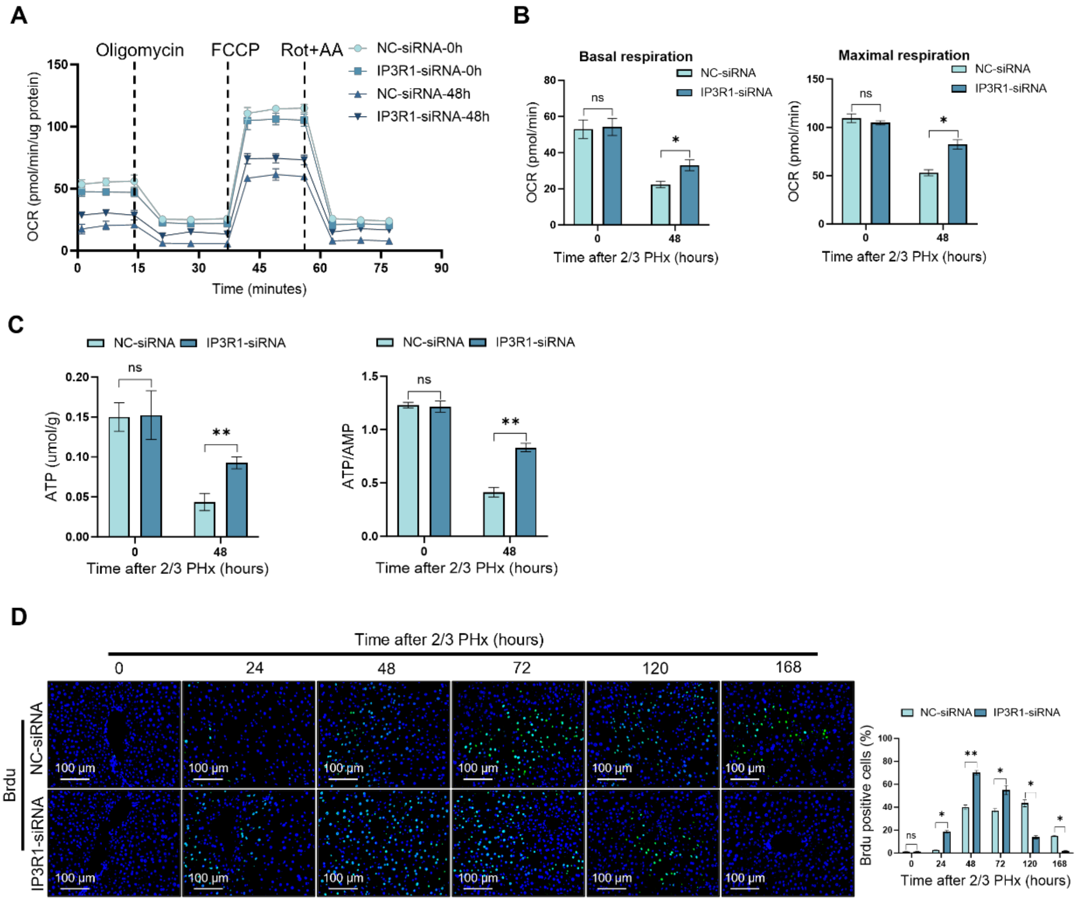

PINK1 inhibits the transport of calcium between the ER and mitochondria to promote liver regeneration. (A) OCR was measured by XF-analyzer; (B) Quantification of OCR; (C) ATP and ATP/AMP level in primary hepatocytes from hKO mice transfected with IP3R-siRNA or NC-siRNA after PHx; (D) Immunofluorescence staining of Brdu in liver tissues from hKO mice transfected with IP3R-siRNA or NC-siRNA post PHx. Data were presented as mean±SEM; n=4–6 per group; $*P < 0.05$, $**P < 0.01$, $***P < 0.001$.
